# Ion distributions around left- and right-handed DNA and RNA duplexes: a comparative study

**DOI:** 10.1093/nar/gku1107

**Published:** 2014-11-26

**Authors:** Feng Pan, Christopher Roland, Celeste Sagui

**Affiliations:** Center for High Performance Simulations (CHiPS) and Department of Physics, North Carolina State University, Raleigh, NC 27695-8202, USA

## Abstract

The ion atmosphere around nucleic acids is an integral part of their solvated structure. However, detailed aspects of the ionic distribution are difficult to probe experimentally, and comparative studies for different structures of the same sequence are almost non-existent. Here, we have used large-scale molecular dynamics simulations to perform a comparative study of the ion distribution around (5′-CGCGCGCGCGCG-3′)_2_ dodecamers in solution in B-DNA, A-RNA, Z-DNA and Z-RNA forms. The CG sequence is very sensitive to ionic strength and it allows the comparison with the rare but important left-handed forms. The ions investigated include Na^+^, K^+^ and Mg^2 +^, with various concentrations of their chloride salts. Our results quantitatively describe the characteristics of the ionic distributions for different structures at varying ionic strengths, tracing these differences to nucleic acid structure and ion type. Several binding pockets with rather long ion residence times are described, both for the monovalent ions and for the hexahydrated Mg[(H_2_O)_6_]^2+^ ion. The conformations of these binding pockets include direct binding through desolvated ion bridges in the GpC steps in B-DNA and A-RNA; direct binding to backbone oxygens; binding of Mg[(H_2_O)_6_]^2+^ to distant phosphates, resulting in acute bending of A-RNA; tight ‘ion traps’ in Z-RNA between C-O2 and the C-O2′ atoms in GpC steps; and others.

## INTRODUCTION

Helices formed by natural amino acids and nucleotides are predominantly right-handed, while left-handed forms, such as PPII helices in proteins and Z-DNA and Z-RNA duplexes, are relatively rare. A left-handed, double-helix DNA with two antiparallel chains joined by Watson-Crick (WC) base pairs was first revealed by a crystal structure of d(CGCGCG)_2_ in 1979 (1). The term ‘Z-DNA’ was coined for this structure because the sugar-phosphate backbone displays a characteristic zig-zag pattern. Z-DNA is formed by dinucleotide repeats, and is a characteristic of sequences that alternate purines and pyrimidines, mainly CG or GC. These kinds of base pairs give rise to an anti–syn alternation, which is due to rotation of the guanine residue around its glycosidic bond, resulting in a syn conformation, while the cytosine retains its anti configuration (2,3). A high density of base sequences favoring Z-DNA is found near transcription start sites (4), where Z-DNA is stabilized by negative supercoiling of DNA (3,5). Z-DNA is induced by a set of binding proteins near promoter regions, which boosts the transcription of downstream genes (6). Z-DNA is highly immunogenic, and antibodies against it (7–9) are used to find locations prone to Z-DNA conformations. The current view is that Z-DNA formation plays a role in gene expression, regulation and recombination (3,6,10–16).

Since the right-handed form is DNA's dominant duplex conformation, research has focused on the microscopic mechanisms behind the B-Z DNA transition and controversial models have ensued (17). Proposed transition mechanisms include: base-pair opening before base-pair plane and phosphate backbone angle rotation within the core of the helix (1); successive flipping of base-pair planes, without any disruption of the WC pairs (18); models with intermediate structure (19–23), such as one with two A-DNA-like intermediates (20); extrusion of bases, as observed in the crystal structure of a B-Z junction (23), followed by propagation and reformation of the pairs. Recent molecular dynamics (MD) simulations indicate that the transition is governed by a complex free energy landscape which allows for the coexistence of several competing mechanisms so that the transition is better described in terms of a reaction path ensemble (24).

After the discovery of Z-DNA, it was found that the right-handed A-RNA double helix made of CG repeats may also be transformed into a left-handed double helix or Z-RNA (25–29) under conditions of high ionic strength or high pressure (30). The first detailed structure of Z-RNA of natural sequence, r(CGCGCG)_2_, was described in a nuclear magnetic resonance (NMR) study at high ionic strength in 2004 (29). However, in contrast to Z-DNA, considerably less is known about Z-RNA and what role it may play in terms of biological functions. Recent experiments—based on binding to the RNA-editing enzyme ADAR1—have probed the structure of Z-RNA under physiological ionic strength conditions, and provided some evidence that there may even be more than one type of Z-RNA present, either *in vitro* or *in vivo* (31).

An important structural determinant of nucleic acids is that they are polyanionic in nature, and water and counterions are crucial for their stability. In solution, counterions surround the nucleic acid structures and neutralize the nucleic acid anionic phosphates. In addition, they can establish water-mediated contacts and less frequent direct contacts with the electronegative groups. These counterions affect both the structure and stability of the nucleic acid conformations, and therefore their biological function. Specifically, counterions can help regulate genome packing (32,33), ribozyme activity (34), RNA folding (35–37), and aid in mediating DNA–protein interactions (38). In fact, due to the highly charged nature of DNA and RNA, it is unlikely that these could be packaged into their compact cellular forms in the absence of counterions (32). Ions also perform an important role in the transition between the right-handed forms at low salt concentrations, and the left-handed forms at high salt concentrations. In solution, the cations move close to the nucleic acid molecule, finding their way into the major and minor grooves, and backbone oxygens. Given the dynamical nature of the system, the cations generally become localized for relatively short periods of time before drifting away and being replaced by other cations from the solution. Similarly, the mobile anions are likely to be excluded from the near nucleic acid region due to electrostatic repulsion. However, nucleotide electropositive edges have been shown to exhibit specific anion binding sites that also turn out to be good locations for the binding of the negatively charged aspartic and glutamic amino acids and negatively charged groups of other ligands (39).

Considering the importance of the ion distribution in stabilizing nucleic acid structure, it is not surprising that this issue has received intense scrutiny over the past three decades. Although the ions are mobile, some of them can be localized long enough (especially divalent cations) to show up as bound ions in X-ray diffraction studies (40–51), although different atomic resolution crystal structures of equal sequences may differ on the presence of bound ions (52,53). Additional improvements have become possible through a combination of anomalous small-angle X-ray scattering (54–56) and atomic emission spectroscopy (57). With these techniques, it has been possible to count explicitly the number of ions in a given region, and thereby provide information as to their time-averaged distributions. NMR studies have also been used to study nucleic acid structure and surrounding ions, especially when precision is improved by the addition of residual dipolar couplings. Thus, for instance, NMR studies have found bound monovalent ions in either the major or minor groove of DNA (42,47,58–63).

However, it is still difficult to obtain true spatial resolution and dynamical information with these experimental techniques. This is where classical MD simulations are extremely useful, as they make it possible to explicitly track the motion of ions around the nucleic acids in order to quantify their locations and effect on structure. In fact, MD simulation of the ion atmosphere around nucleic acids has been around for decades (39,64–79), especially since the correct treatment of electrostatics (80–84) led to stable, reliable trajectories (81). With some exceptions, the majority of the work has focused on B-DNA. Only recently have more systematic studies on A-RNA emerged (85–89). In addition, much effort in the refinement of nucleic acid fields has taken place in the last decade (79,90–92), leading to more accurate results in the relatively long-time simulations that are required for the equilibration of the ion distribution.

In this article, we report on a large-scale MD study of the ion distribution around individual (5′-CGCGCGCGCGCG-3′)_2_ dodecamers in solution in B-DNA, A-RNA, Z-DNA and Z-RNA forms. The duplexes are immersed in rather large water boxes to account for the fact that ionic distribution functions need to be calculated to ∼30 Å. The study involves 20 simulations lasting 120 ns each. We chose this sequence because we wanted to carry out a comparative study of ion distribution, and the CG sequence is crucial for the left-handed forms. A fair comparison between the different structures needs the same sequence, as previous simulations have shown that the ion distributions exhibit sequence-dependent features. From a chemical point of view, a comparison between RNA and DNA structures only involves the presence or absence of the 2′-OH group in the sugar (and avoids the extra T/U change associated with AT sequences). The regularity of the sequence also allows for much more ‘clear-cut’ results and conclusions about distribution around CG pairs. Salt effects in this sequence have been studied in 2000, via 2.5-ns long simulations involving the distribution of the K^+^ ion (70); and more recently in simulations that studied the effect of force fields, water model and salt concentration on the structure of A-RNA (85,87) (these studies did not report results on the ionic distribution itself). Other than that, most of the recent simulations on the ion atmosphere around right-handed nucleic acid duplexes employ sequences that are relatively rich in A and T or U nucleotides. Although *in vitro* the transition from the right-handed forms to the left-handed forms can be triggered with the addition of salt at high concentrations (29), the Z-forms also exist under physiological ionic strength (31). Simulations allow us to stabilize the left-handed forms and then to slowly increase the concentration of salt in order to discern how ion binding is linked to the structure of the duplex.

In terms of ions, we investigated the distribution of the monovalent Na^+^ and K^+^, and divalent Mg^2+^ ions around these structures, with various concentration values of their chloride salts. The cations most frequently found around nucleic acids are K^+^ (∼0.14 M inside the cell) and Mg^2+^, while the Na^+^ ion is most frequently found in extracellular fluids (76). Since most studies of monovalent ions around nucleic acids involve the Na^+^ ion, it is of interest to study the differences in binding between Na^+^ and K^+^. In this work, we carry out a comparative study of the distributions of each of these cations around each of the four possible duplexes and discuss in detail the sources of the various, important observed differences.

## MATERIALS AND METHODS

Much effort in the refinement of nucleic acid force fields has taken place in the last decade (79), including quite recent reparameterizations in the AMBER force field (90–92). In this work, large-scale MD simulations were used to explore the ion distribution around DNA and RNA sequences (CG)_6_ (a shorthand for (5′-CGCGCGCGCGCG-3′)_2_) in an explicit solvent environment. The simulations were carried out using the PMEMD module of the AMBER 12 (93) software package with the ff12SB force field with parameters ff99BSC0 (90) for DNA and ff99BSC0+χ_OL3_ (87,91,92) for RNA. The TIP3P model (94) was used for the water molecules. The duplexes were placed in cubic box of ∼82 Å side, filled with a suitable number of water molecules. Such a large box is necessary, since cylindrical distribution functions (CDFs) need to be calculated to a distance of at least 30 Å. Simulation details and the equilibration process, which took longer than 1.5 ns, are given in the Supporting Information (SI) associated with this paper. The equilibration process was followed by 120 ns of constant pressure and temperature production runs. A system of oligonucleotides, water and ions takes long to reach full equilibrium, and the motion of ions is the rate-determining step for convergence (72). Indeed, for a palindromic sequence of a DNA dodecamer, it has been shown that convergence of the ion distribution in each strand (so that it reflects the symmetry of the sequence) takes ∼100 ns (73) (although internal structural parameters can take shorter times, anywhere between 10 and 50 ns (87)). Thus, equilibrium data were collected from only the last 100 ns of these runs, since a minimum of 20 ns is required in order to stabilize the ion distribution.

Five simulations were carried out for each duplex with the following ions: (i) 22 neutralizing Na^+^, no excess salt; (ii) 22 neutralizing Na^+^ and 0.4-M NaCl; (iii) 22 neutralizing K^+^ and 0.4-M KCl; (iv) 11 neutralizing Mg^2+^ and 0.2-M MgCl_2_; and (v) 22 neutralizing Na^+^ and 4.0-M NaCl. Here, the ‘salt’ molarity is used to indicate ‘excess’ salt (over the neutralizing ions). Thus, we will refer to the case (i) above as ‘zero salt’, although it has 0.06-M Na^+^, due to the neutralizing ions. Ions parameters are given in the SI.

Our analysis was focused on calculating the distribution of the mobile ions around the nucleic acid structures. To that end, we calculated the diffusion coefficients for the ions, the cylindrical and radial distribution functions (RDFs), and carried out an analysis of the efficacy of different sites in localizing the ions. The definitions for these quantities are standard, and their details are given in the SI section.

**Figure 1.**
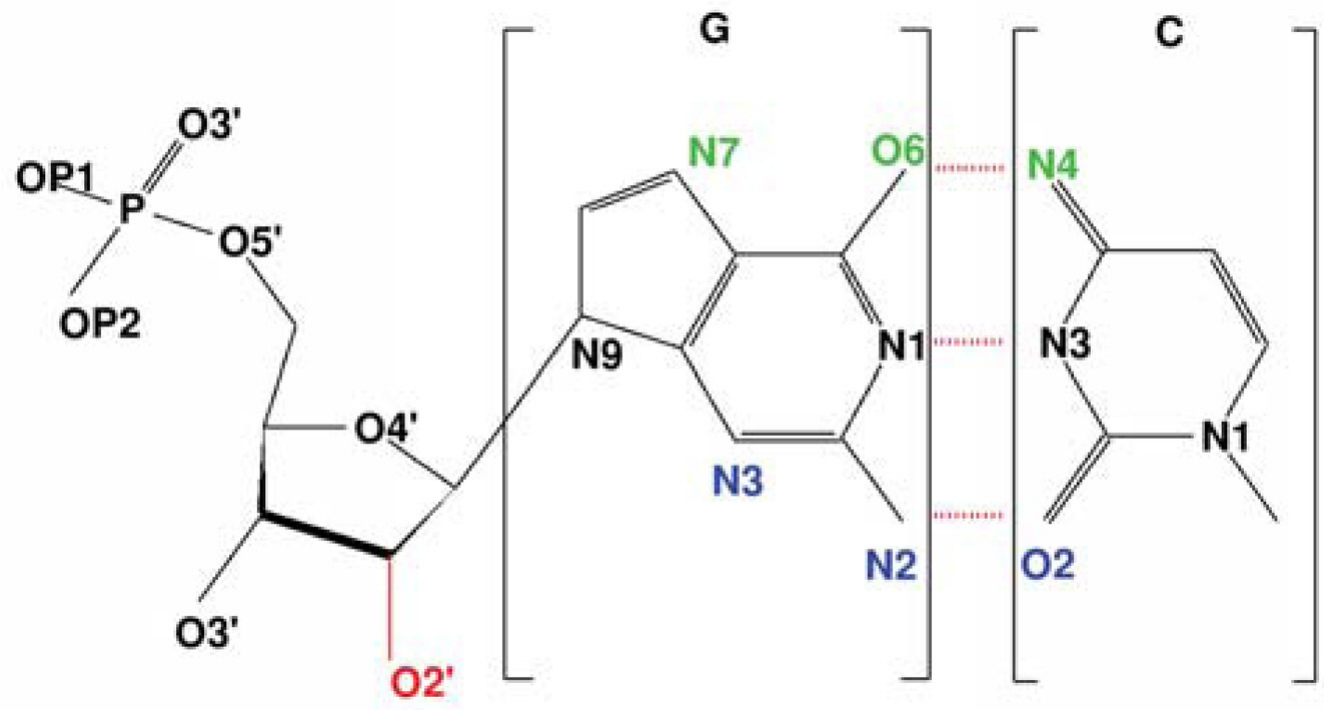
Atomic depiction of a CG Watson–Crick pair. Green and blue atoms represent the important major and minor groove atoms responsible for ion localization. The oxygen O2′ atom in RNA is marked in red. Hydrogen atoms are omitted for clarity.

In terms of an analysis of a potential binding site, a Na^+^ (K^+^) ion was considered to be ‘bound’ to or localized next to a specific atom on the duplex if its distance to that atom was less than 3 Å (3.5 Å) for direct binding, or less than 6 Å when mediated by intervening water molecules. The direct binding distance corresponds to a minimum in the RDF around the electronegative nucleic acid atoms, which separates the first solvation shell from the second solvation shell. Both base and backbone sites were considered when calculating the occupancies. Occupancy was defined as the percentage of time that at least one ion was bound to a given duplex atom during the data collection time of the simulation (last 100 ns). An ion can potentially contribute to the occupancy of more than one atom in the duplex. For B-DNA and A-RNA, C(O2,N1) and G(N2,N3,N9) belong to the minor groove and C(N4) and G(O6,N7) belong to the major groove (Figure 1). The situation is less clear for the left-handed forms. For instance, in Z-DNA the minor groove is clearly discernible, while the major groove becomes flat. Here, we will use the same atomic conventions for the major and minor grooves as for the right-handed forms, as this convention has been used previously in the literature for Z-DNA (1) and for Z-RNA (29). For the backbone, we consider the phosphate oxygens, OP1 and OP2, the phosphate esteric oxygens, O5′ and O3′, the sugar-ring oxygen, O4′, and for the RNA structures the 2′-OH oxygen, O2′ (we will refer to these collectively as the O′ oxygens).

The interaction between duplex sites and ions is, of course, a dynamical affair. Thus, an ion at a given binding site may drift away and then, after a period of time, be replaced by a new ion. Hence, to quantify how long an ion stays in a given binding site, we define a residence time *τ*_*R*_ by means of a standard time correlation function *C*(*t*)_*i*_ for binding site *i*:

\begin{equation*} C(t)_i=\sum _{t_o}\sum _tp_i(t_o)p_i(t_o+t), \end{equation*}

where *p*_*i*_(*t*) is unity if the site is occupied by an ion within 3 Å for Na^+^ and 3.5 Å for K^+^, and zero otherwise. Typically, this function takes the form of a decaying exponential and so *C*(*t*)_*i*_ ∼ exp(*−t*/*τ*_*R*_) which in turn defines the residence time *τ*_*R*_ as a time constant. In our simulations, *C*(*t*)_*i*_ were measured over time intervals of 10 ps to 1 ns, with an increasing step of 10 ps.

## RESULTS

### Cylindrical distribution functions

**Figure 2.**
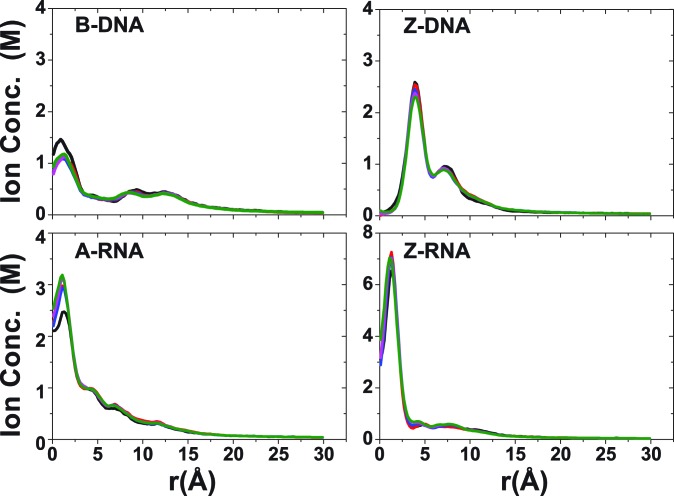
CDFs for Na^+^ ions at zero salt. These plots also illustrate the convergence of these CDFs over time with different colors indicating ever increasing time intervals: black (20–30 ns), red (20–40 ns), blue (20–50 ns), magenta (20–60 ns) and green (20–70 ns).

**Figure 3.**
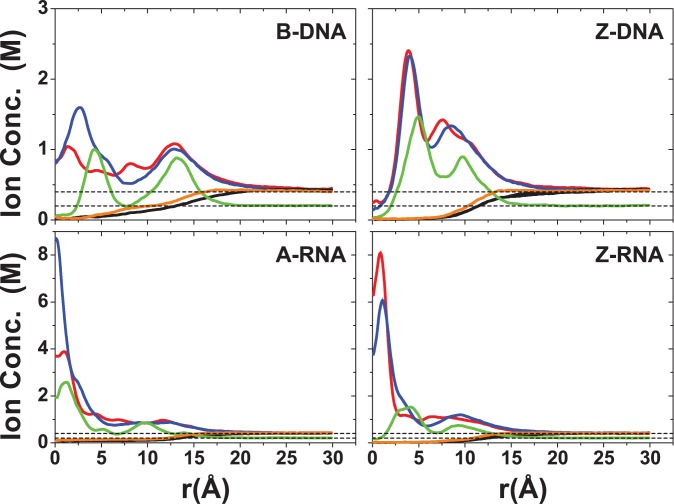
Converged CDFs at high salt concentrations. CDFs are shown for the three cations: Na^+^ (red), K^+^ (blue), Mg^2+^ (green); and negative ions: Cl^−^ (black) for NaCl and KCl, and Cl^−^ (orange) for MgCl_2_. Results are under conditions of high salt concentration: 0.4 M for NaCl/KCl and 0.2 M for MgCl_2_ (plus the corresponding neutralizing cations). Results for Cl^−^ ions for NaCl/KCl are virtually identical, and so these are not color differentiated.

Figures 2 and 3 illustrate the CDFs for the different duplex structures at low and high salt concentrations, respectively. Figure 2 shows convergence of the CDFs, which occurs around 20 ns in the left-handed forms and after 40 ns in the right-handed forms. At zero-salt concentration (with only neutralizing Na^+^; Figure 2), the peaks associated with the RNA structures are higher than those for the corresponding DNA structures, indicating that the number of ions localized around the RNA structures is larger than that for the DNA counterparts. In addition, the peaks associated with the left-handed structures are higher than those for the corresponding right-handed structures, pointing to higher localization of ions around the left-handed structures. The fall-off from the first peak differs considerably between the structures. For B-DNA, this is characterized by a set of minima and maxima between 7 and 14 Å, followed by a smooth decay to approximately zero (<0.05 M, which is the concentration of 22 Na^+^ ions in the given box) at ∼28 Å. By contrast, Z-DNA has a single secondary maximum between 6 and 8 Å followed by a smooth decay to ∼0 at ∼23 Å. For A-RNA, there are a few, shallow oscillations after the initial peak, and it takes ∼26 Å to decay to zero. Likewise, a set of very shallow minima and maxima characterizes the Z-RNA fall-off, followed by a smooth decay to zero at ∼25 Å.

Turning to the results for high salt ( 22 Na^+^ ions + 0.4-M NaCl; 22 K^+^ ions + 0.4-M KCl; 11 Mg^2+^ ions + 0.2-M MgCl_2_) in Figure 3, B-DNA now has a number of well discernible peaks associated with each of the three cations, Na^+^, K^+^ and Mg^2+^. By comparison to the zero-salt distribution of Na^+^ ions in Figure 2, there are now several lower peaks for Na^+^ before the distribution smoothly decays to the bulk concentration value. The distributions for the three cations around B-DNA indicate the presence of two equally important ‘binding shells’ (more diffuse in the case of Na^+^). The second binding shell is also present in Z-DNA, although its peak is considerably lower than the first peak. Distributions for Na^+^ and K^+^ ions are similar for Z-DNA, but the first peak is much more prominent for K^+^ than Na^+^ ions in B-DNA and A-RNA, while the first Na^+^ peak is higher than the K^+^ one for Z-RNA. In the RNA duplexes, the ions can become very close to the central axis. The peaks associated with the Mg^2+^ ions are shifted outward, in comparison to the peaks associated with the monovalent cations (except for A-RNA where the peaks for Na^+^ and Mg^2+^ ions are centered at approximately the same distance).

**Figure 4.**
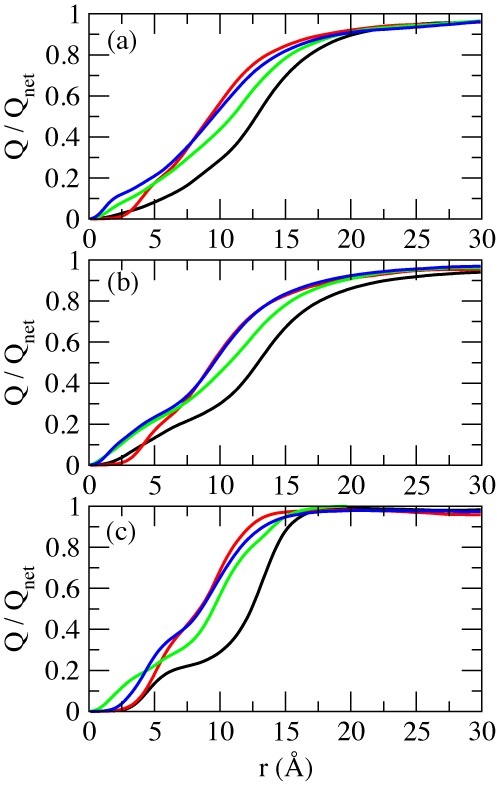
Accumulation of total ionic charge (counterions + co-ions) as a function of radial distance from the central axis of the duplex. The values are normalized with respect to the net charge of the duplex sequence (−22e). Different colors represent different structures: B-DNA (black); Z-DNA (red); A-RNA (green); Z-RNA (blue). (**a**) Na^+^ at 0.4-M NaCl; (**b**) K^+^ at 0.4-M KCl; (**c**) Mg^2+^ at 0.2-M MgCl_2_.

The total ionic charge (both co-ions and counterions) accumulated as a function of radial distance from the helical axis is shown in Figure 4. The charge is normalized with respect to the total charge of the nucleic acid duplex (−22e). For any given distance before the asymptotic value, A-RNA is more screened than B-DNA. For regions close to the axis, A-RNA localizes more Na^+^ and K^+^ ions than both B-DNA and Z-DNA, and more Mg^2+^ ions than the three other forms. At intermediate distances, charge neutralization works better in the left-handed forms (which are comparable). The Mg^2+^ ion distribution is considerably more localized than the Na^+^ ion distribution, which in turn is slightly more localized than the K^+^ ion distribution.

### Radial distribution functions

**Figure 5.**
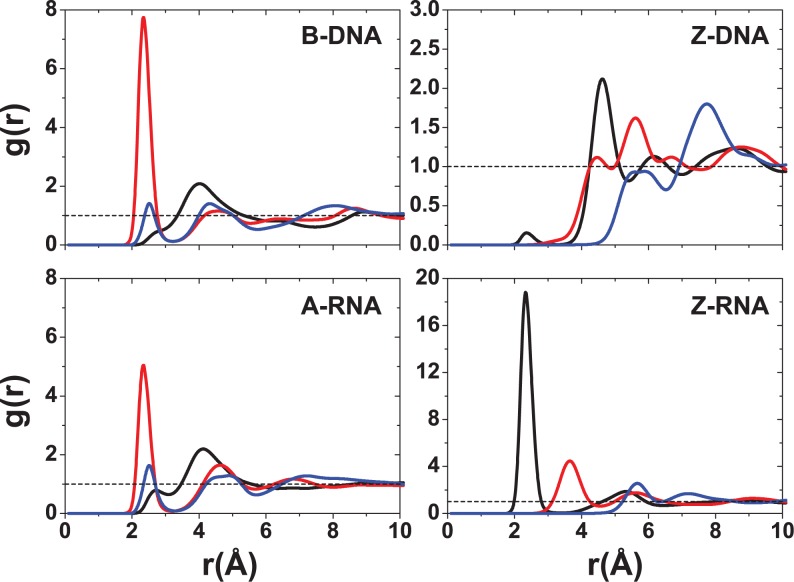
RDFs for Na^+^ with respect to major or minor groove atoms at 0.4-M salt concentration. For B-DNA and A-RNA, the colors indicate RDFs with respect to atoms in the major groove: O6 (red), N7 (blue) and N4 (black). For Z-DNA and Z-RNA, the colors indicate RDFs with respect to atoms in the minor groove: O2 (black), N2 (red) and N3 (blue).

**Figure 6.**
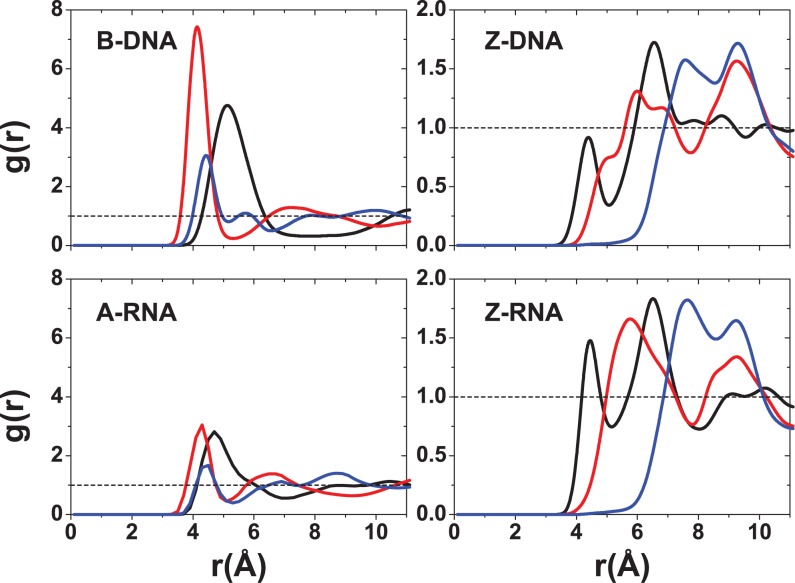
RDFs for Mg^2+^ with respect to major or minor groove atoms at 0.2-M salt concentration. For B-DNA and A-RNA, the colors indicate RDFs with respect to atoms in the major groove: O6 (red), N7 (blue) and N4 (black). For Z-DNA and Z-RNA, the colors indicate RDFs with respect to atoms in the minor groove: O2 (black), N2 (red) and N3 (blue).

**Figure 7.**
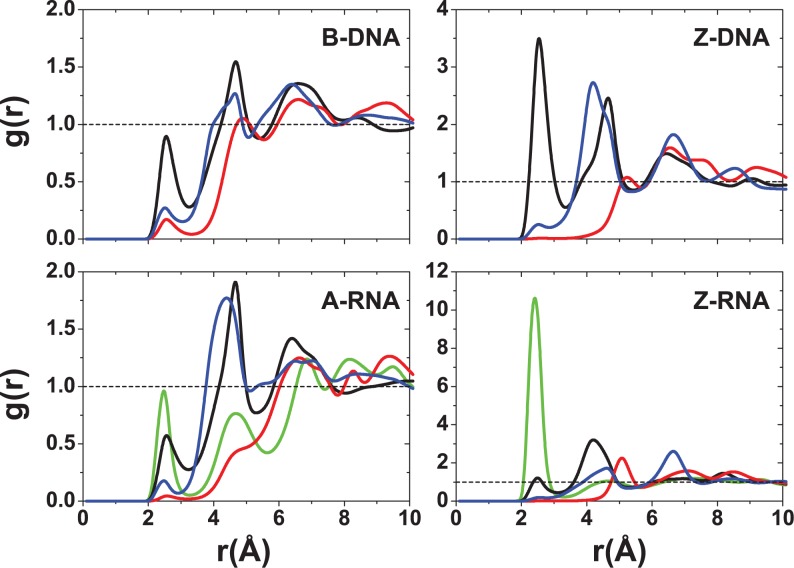
RDFs for Na^+^ with respect to O′ backbone oxygens at 0.4-M salt concentration. Colors indicate: O2′ (green), O3′ (black), O4′ (red) and O5′ (blue).

**Figure 8.**
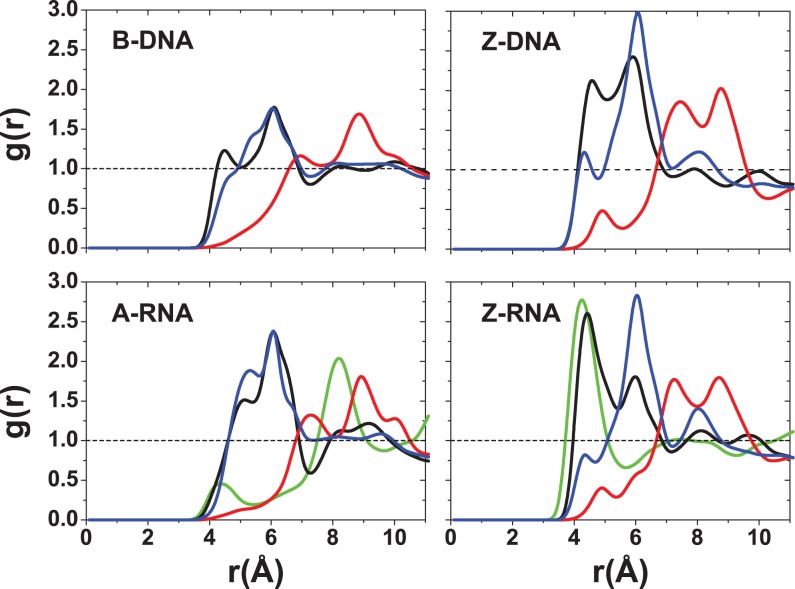
RDFs for Mg^2+^ with respect to O′ backbone oxygens at 0.2-M salt concentration. Colors indicate: O2′ (green), O3′ (black), O4′ (red) and O5′ (blue).

**Figure 9.**
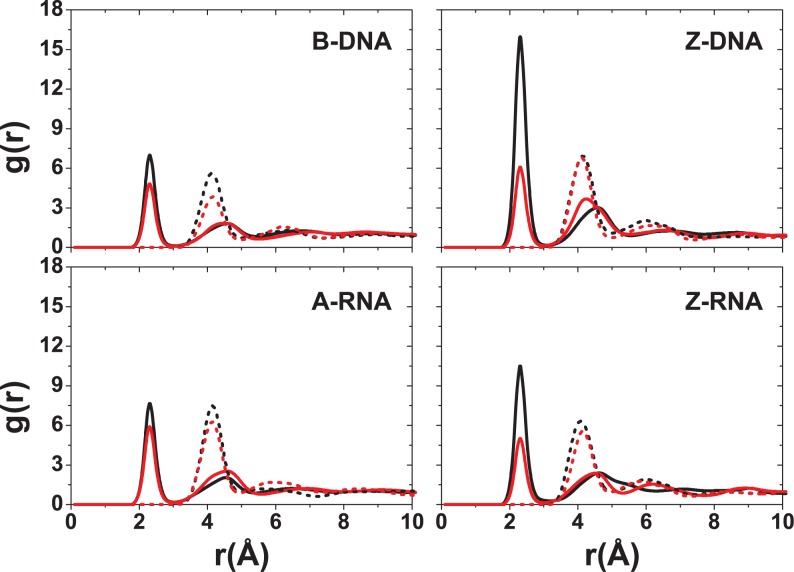
RDFs for Na^+^ (solid lines, 0.4-M salt concentration) and Mg^2+^ (dashed lines, 0.2-M salt concentration) with respect to phosphate oxygens. Colors indicate: OP1 (black) and OP2 (red).

Figures 5–9 display RDFs of the Na^+^ and Mg^2+^ ions with respect to different DNA/RNA atoms. Within the major groove, there are qualitative similarities for Na^+^ binding between B-DNA and A-RNA in Figure 5. The first peak of the electronegative O6 atom occurs at 2.4 Å for both B-DNA and A-RNA. The bulky, hydrated Mg^2+^ (Figure 6) is displaced outward with binding distances with respect to O6 of 4.1 Å (B-DNA) and 4.3 Å (A-RNA). For the left-handed forms, differences in Na^+^ binding are considerable. In particular, Z-RNA displays far stronger binding than Z-DNA, characterized by very strong O2 binding (at 2.3 Å), while Z-DNA has relatively little direct binding to O2 at 2.4 Å and a second more important, indirect binding peak at 4.6 Å. The distributions of Mg^2+^ for the Z forms seem to indicate mainly indirect binding and are qualitatively more similar.

Figure 7–9 show the RDFs between the ions and the oxygen atoms in the backbone. Figure 7 for the Na^+^ ion shows a qualitative similarity between the B-DNA and A-RNA binding, with additional binding by the O2′ atom in RNA. The left-handed forms, on the other hand, show striking differences. Z-DNA has a strong peak for O3′ at 2.6 Å and important secondary binding at 4.7 Å for O3′ and at 4.2 Å for O5′. Z-RNA has important indirect binding for O3′ at 4.2 Å but in addition it presents a dominant peak for O2′ at 2.4 Å. For Mg^2+^, the distributions are relatively more similar (with stronger binding for the RNA forms; Figure 8) except, of course, for the binding to O2′ in the RNA forms. The RDFs for binding with the phosphate oxygens (OP1,OP2) are shown in Figure 9. For a given ion, the RDFs for the different structures all resemble each other closely, with stronger binding in the Z forms. For Na^+^, OP1 binding is preferred over OP2, especially in the Z forms. Direct binding of Na^+^ to the four duplexes takes place at ∼2.3 Å, and a weaker, secondary binding occurs at 4.5 Å. For Mg^2+^ ions, the binding is all indirect (alternatively, it is direct binding of the hexahydrated Mg[(H_2_O)_6_]^2+^ ion). The RDFs for K^+^ ions are given in the SI section. In general, K^+^ seems to be more tightly bound than Na^+^ in the right-handed forms, and comparably in the Z forms (except for a large peak for binding of Na^+^ to O2 in Z-RNA).

### Ion binding as a function of sequence

**Figure 10.**
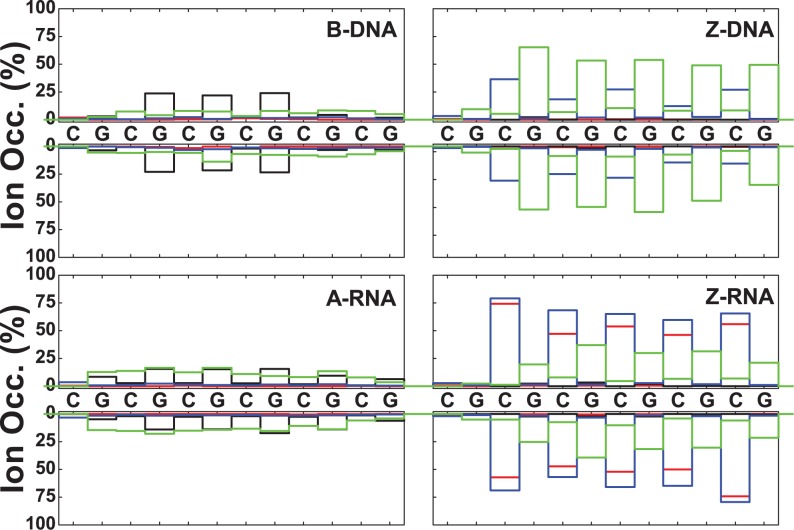
Na^+^ ion occupancies within 3 Å as a function of sequence for zero salt concentration. Colors represent: major groove (black), minor groove (red), O′ oxygen atoms on backbone (blue) and phosphate oxygens (green).

**Figure 11.**
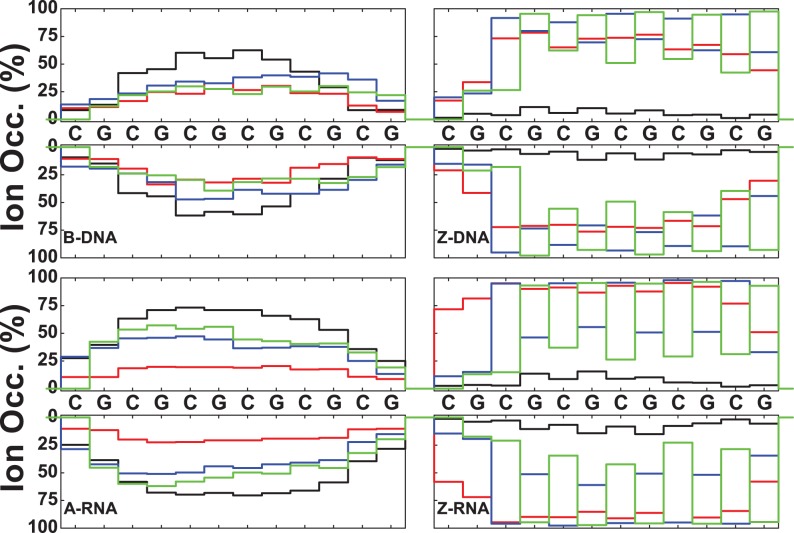
Na^+^ ion occupancies within 6 Å as a function of sequence for zero salt concentration. Colors represent: major groove (black), minor groove (red), O′ oxygen atoms on backbone (blue) and phosphate oxygens (green).

In addition to the RDFs, we have investigated ion binding to specific DNA/RNA atoms in a number of ways. Figures 10 and 11 plot the ion occupancy of each nucleotide in the sequence: the top part describes the 5′–3′ strand from left to right, while the lower part gives the occupancy of the other strand in reverse order (i.e. also in the 5′–3′ from left to right). This way of displaying the information should show mirror symmetry with respect to the horizontal axis upon ion distribution convergence. Figure 10 plots the occupancy results for Na^+^ ions close to the nucleotides (i.e. within 3 Å) for zero salt concentration (just neutralizing Na^+^ ions). Figure 11 shows similar results for ion occupancies within 6 Å, and therefore includes not only the so-called direct bindings but also the Na^+^ ion bindings mediated by approximately a single water molecule. In terms of the direct binding results, Figure 10 shows that for B-DNA most of the ion binding with ∼25% occupancy is associated with three central G nucleotides of the major groove, while the minor groove and backbone O binding are either very small or negligible. For A-RNA, the major groove binding becomes distributed among all the G nucleotides with occupancies of ∼15%; and the binding with the phosphate oxygens, in the 10–15% range, is distributed more or less uniformly along the backbone. By contrast, the phosphate oxygen binding in Z-DNA is strongly centered on the Gs with populations around 50–60%; binding to the O′ oxygens is also important, in the range 20–40%, and centered on the Cs; and binding to the major and minor grooves is negligible. Z-RNA also presents binding to the phosphate oxygens on the Gs and to the O′ oxygens on the Cs, but their relative importance is reversed compared to Z-DNA: with ranges 20–40% for the phosphate oxygens and ranges 60–80% for the O′ oxygens. Binding to the minor groove along the Cs in Z-RNA is also very important, with ranges 50–70%. The net effect of including ion binding all the way to 6 Å is illustrated in Figure 11. Naturally, the occupancy becomes much larger and more uniform. Results for the high salt concentration (NaCl and KCl) and Mg^2+^ ions are presented in the SI section.

**Figure 12.**
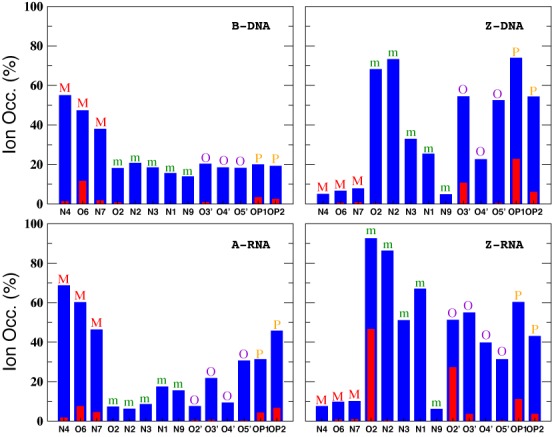
Na^+^ ion occupancies with respect to specific atoms at zero salt concentration. Occupations for direct and indirect binding are indicated with red (3 Å) and blue (6 Å). The results represent average values with respect to the entire duplex. Letters on top of the bars represent different nucleic acid regions: M (major groove), m (minor groove), O (O′ oxygen atoms on backbone) and P (phosphate oxygens).

**Figure 13.**
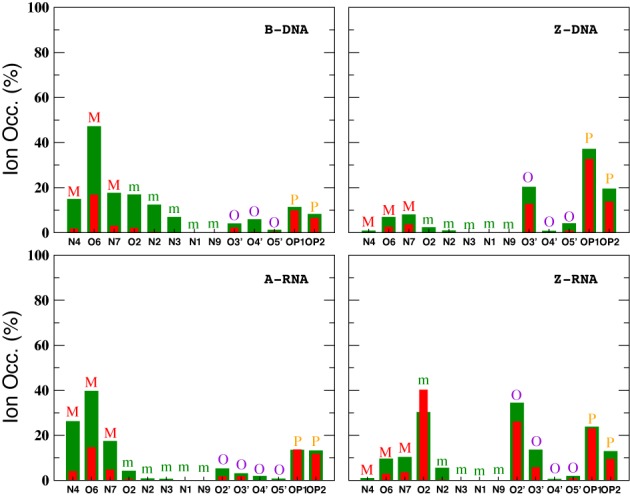
Ion occupancies with respect to specific atoms at 0.4-M salt concentration within the direct binding region. Colors represent ion type: Na^+^ (red) and K^+^ (green). The results represent average values with respect to the entire duplex. Letters on top of the bars represent different nucleic acid regions: M (major groove), m (minor groove), O (O′ oxygen atoms on backbone) and P (phosphate oxygens).

In addition to the different distribution functions already discussed, it is possible to further characterize the localization of ions due to particular atoms in the nucleic acid duplexes. Figure 12 gives bar plots for the Na^+^ ion occupancies for direct (≤3 Å) and indirect (≤6 Å) binding with respect to specific atoms at zero salt. In agreement with the CDFs and RDFs presented before, there is similarity between the right-handed structures for the Na^+^ ions, with the O6 atom contributing more to direct binding (the OP oxygens have a comparable contribution for A-RNA). The left-handed structures, on the other hand, differ considerably with respect to each other and with respect to their right-handed counterparts. Figure 12 shows that most direct binding in Z-DNA occurs at OP1 with secondary contributions from OP2 and O3′. By contrast, in Z-RNA there is a big contribution to binding by O2 (see also Figure 5), followed by O2′ and, to a lesser degree, the phosphate oxygens and O3′. Figure 13 compares direct binding for K^+^ and Na^+^ (for 0.4-M salt concentration). In general, K^+^ exhibits larger ion occupancy, especially in the major groove for the right-handed forms (and part of the minor groove for B-DNA). The only exception to this observation occurs at atom O2 in Z-RNA, where Na^+^ exhibits higher ion occupancy. Comparing direct binding for Na^+^ in Figure 12 (zero salt) and Figure 13 (0.4-M salt concentration), one can see that there are only relatively small changes in the ion occupancies, indicating that these sites are primarily saturated at low concentrations.

### Anion distribution

The aim of this work is to characterize the cation distribution around the four types of duplexes. However, we also checked whether the Cl^−^ anions had contacts with the nucleic acids. We found that, indeed, there were short-lived contacts. For instance, a contact close to C-N4 was seen in all four duplexes, but its occupation was extremely low. In addition, we found low-population bindings to the O2′ sugar groups for the RNA duplexes, and to G-N2 in B-DNA and A-RNA. So indeed, it is possible for the anion to intrude the first hydration shell of nucleic acids (39), but these events are too rare to be statistically significant. The CDFs in Figure 3 show that the behavior of the Cl^−^ anion distribution is similar in all cases: it is close to zero next to the helical axis with a smooth increase out to its bulk value. For the DNA structures, values of the Cl^−^ distribution start to rise before 10 Å, while for the RNA structures the values start to rise only after 10 Å: Cl^−^ anions generally are not allowed near the duplexes, and this repulsion is stronger for the RNA structures.

### Structural interpretation of ion occupation

**Figure 14.**
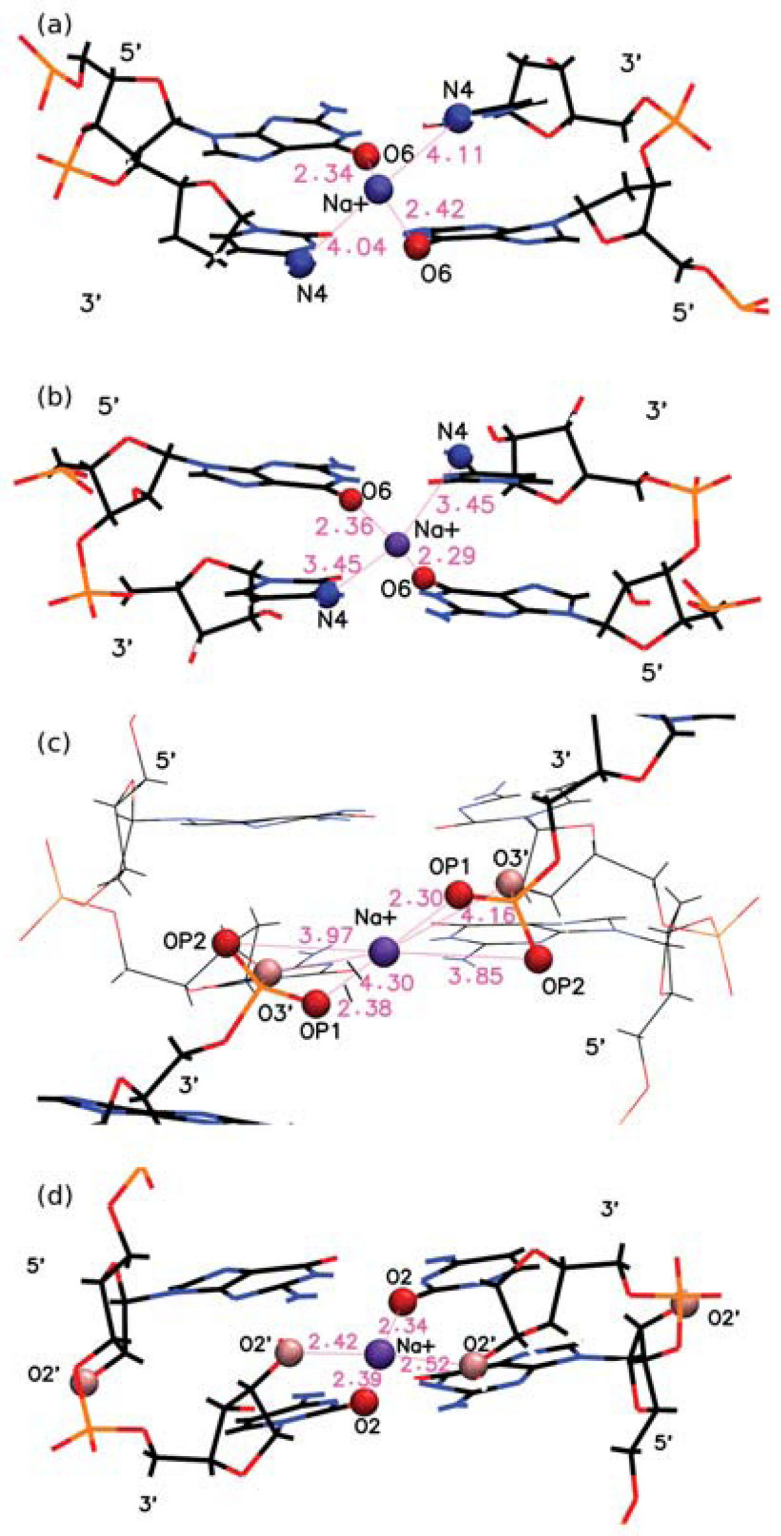
Atomistic details of some direct binding sites of Na^+^ for different nucleic acid structures. (**a**) Binding to G-O6 (and proximity to C-N4) in B-DNA; (**b**) binding to G-O6 (and proximity to C-N4) in A-RNA; (**c**) binding to G-OP1, G-OP2 and C-O3′ in Z-DNA; (**d**) binding to C-O2 and C-O2′ in Z-RNA. The configurations shown are just a snapshot at a given time obtained from the MD simulations. Details of (a) are also provided in Figure S8.

Table 1,3 gives the residence times for the three ions, and Table 2 gives the average distances of the monovalent ions to the nucleotide atoms. Figure 14 illustrates some typical binding pockets for the Na^+^ ion in the different nucleic acid structures. Figure 14a and b gives a snapshot of the binding of the Na^+^ ion by O6 in B-DNA and A-RNA. The ion is directly bound to the O6 atoms and also close to the N4 atoms of the major groove. This binding pocket is shown in more detail in Figure S8, where the hydrogen atoms covalently linked to the N4 atoms and four bound waters are depicted explicitly. In this conformation, the Na^+^ ion is strongly bound by the G-O6 atoms, and given the geometry of the GpC steps, the ion manages to position itself relatively close to the N4 atoms by avoiding the electropositive N4 hydrogen atoms, which point away from the ion. In the process, the ion loses two of the waters in its binding shell. In A-RNA, the distance to the N4 atoms decreases slightly, as shown in Table 2. This is particularly true for K^+^, which shows a residence time of 2.4 ns for N4. In the four cases (B-DNA/A-RNA combined with Na^+^/K^+^), the configurations can be described as ion bridges, without intervening waters between the ions and the O6 and N4 atoms. In addition, K^+^ exhibits long residence times for O2 and N2 in the minor groove of B-DNA. Figure 14c shows the direct binding of Na^+^ to the phosphate oxygens in a CpG step in Z-DNA and Figure 14d shows the binding of Na^+^ to O2 and O2′ in Z-RNA. In Z-DNA, the binding of Na^+^ to G-OP1 is direct, with the longest residence time (∼2.3 ns) and a short binding distance of ∼2.4 Å. Instead, the OP1 in the C nucleotide points outside the helical core in Z-DNA, and therefore its residence time is only ∼0.43 ns. With respect to the OP2 atoms in Z-DNA, the residence time of Na^+^ is ∼0.35 ns in C-OP2 and 0.65 ns in G-OP2. For the Z-RNA conformation shown in Figure 14d, the cation is localized by four atoms: two each of O2 and O2′ all situated in the C nucleotides. These atoms are relatively close to the cation, with distances in the 2.3–2.5 Å range. This illustrates how Z-RNA is much more efficient in localizing or trapping cations, and is reflected in the residence times given in Table 1. The times associated with Z-RNA O2 in C and O2′ in C are very high: 8.6 ns and 7.8 ns, respectively. These residence times are so much longer than the times associated with other binding sites that ions making their way into these positions in Z-RNA are effectively trapped there for a very long time. We have also examined the characteristics of the OP1 and OP2 binding for Z-RNA, which qualitatively resemble those of Z-DNA.

**Table 1.** Residence time (in ns) of Na^+^ and K^+^ for different atoms in the nucleic acid structures

	Na^+^(K^+^) residence times (ns)									
B-DNA	N4	O2	O6	N2	N3	N7	O3′	O4′	OP1	OP2
	0.83(1.74)	–(1.82)	1.01(2.03)	–(1.84)	–(1.48)	0.25(1.37)	0.38(0.41)	–(1.74)	0.39(0.86)	0.35(0.80)
Z-DNA	O2	O6	N7	O3′(C)	O3′(G)	OP1(C)	OP1(G)	OP2(C)	OP2(G)	
	–(0.30)	–(0.62)	–(0.68)	1.25(2.12)	–(0.51)	0.43(1.18)	2.26(4.75)	0.35(0.86)	0.65(1.84)	
A-RNA	N4	O2	O6	N7	O2′	OP1	OP2			
	0.43(2.45)	–(0.38)	0.54(1.47)	0.35(0.85)	–(0.52)	0.42(1.58)	0.47(1.56)			
Z-RNA	O2	O6	O2′(C)	O2′(G)	O3′(C)	O3′(G)	OP1(C)	OP1(G)	OP2(C)	OP2(G)
	7.83(2.79)	–(0.91)	8.56(5.42)	–(0.71)	1.10(1.43)	–(0.67)	0.43(0.99)	1.21(1.92)	0.36(0.76)	0.60(1.82)

These results are for 0.4-M salt concentration.

**Table 2.** Distance from ions to specific nucleic acid atoms for direct binding

	Na^+^(K^+^) distances (Å)					
B-DNA	O6(G6)	O6(G18)	N4(C7)	N4(C19)		
	2.38 ± 0.13(2.78 ± 0.17)	2.40 ± 0.14(2.80 ± 0.19)	3.72 ± 0.50(3.87 ± 0.56)	3.58 ± 0.54(4.06 ± 0.61)		
Z-DNA	OP1(G8)	OP1(G20)	OP2(G8)	OP2(G20)	O3′(C7)	O3′(C19)
	2.39 ± 0.15(2.82 ± 0.20)	2.36 ± 0.13(2.80 ± 0.19)	3.86 ± 0.36(4.11 ± 0.39)	4.12 ± 0.30(4.13 ± 0.41)	3.96 ± 0.26(3.39 ± 0.54)	3.97 ± 0.27(3.37 ± 0.52)
A-RNA	O6(G6)	O6(G18)	N4(C7)	N4(C19)		
	2.36 ± 0.11(2.79 ± 0.17)	2.39 ± 0.14(2.78 ± 0.18)	3.13 ± 0.52(3.54 ± 0.36)	3.59 ± 0.38(3.66 ± 0.40)		
Z-RNA	O2(C7)	O2(C19)	O2′(C7)	O2′(C19)		
	2.39 ± 0.13(2.83 ± 0.21)	2.39 ± 0.12(2.79 ± 0.18)	2.43 ± 0.13(2.80 ± 0.15)	2.44 ± 0.13(2.77 ± 0.14)		

The labels in the parenthesis refer to the residues to which the atoms belong. Usually four to six atoms act to form a single ion trap in the middle part of a duplex, as shown in Figure 14. The distances are averages and are based on the last 10 ns of MD simulations.

**Table 3.** Residence time (in ns) of Mg^2+^ for different atoms in the nucleic acid structures

	Mg^2+^ residence times (ns)														
B-DNA	N4	O6	N7	O3′	O5′	OP1	OP2								
	2.04	9.43	2.21	0.79	0.52	1.20	0.93								
Z-DNA	O2	O6	N2	N7	O3′(C)	O3′(G)	O5′(C)	O5′(G)	OP1(C)	OP1(G)	OP2(C)	OP2(G)			
	1.08	0.57	1.04	0.59	2.61	0.56	1.37	1.15	2.15	7.00	1.24	5.62			
A-RNA	N4	O6	N7	O2′	O3′	O5′	OP1	OP2							
	2.20	2.42	1.13	0.42	0.75	0.82	2.25	2.10							
Z-RNA	N4	O2	O6	N2	N7	O2′(C)	O2′(G)	O3′(C)	O3′(G)	O5′(C)	O5′(G)	OP1(C)	OP1(G)	OP2(C)	OP2(G)
	0.71	1.89	0.90	1.02	0.96	6.04	0.57	4.93	0.48	0.96	0.95	1.73	7.61	0.85	4.99

These results are for 0.2-M salt concentration, using a cutoff of 5 Å.

Residence times associated with K^+^ are considerably longer than those for Na^+^, except for Z-RNA where Na^+^ has longer residence time for the O2 and O2′ in C. The binding distances for K^+^ tend to be longer than those for Na^+^, reflecting the larger size of the K^+^ ion (for which we considered distances ≤3.5 Å as direct binding). An exception to this is seen in cytosine O3′ in Z-DNA which displays direct binding for K^+^ (residence time of 2.1 ns) but not for Na^+^. Smaller fluctuations in the binding distances shown in Table 2 correlate with stronger, direct binding, while larger fluctuations correlate with higher mobility and indirect binding.

**Figure 15.**
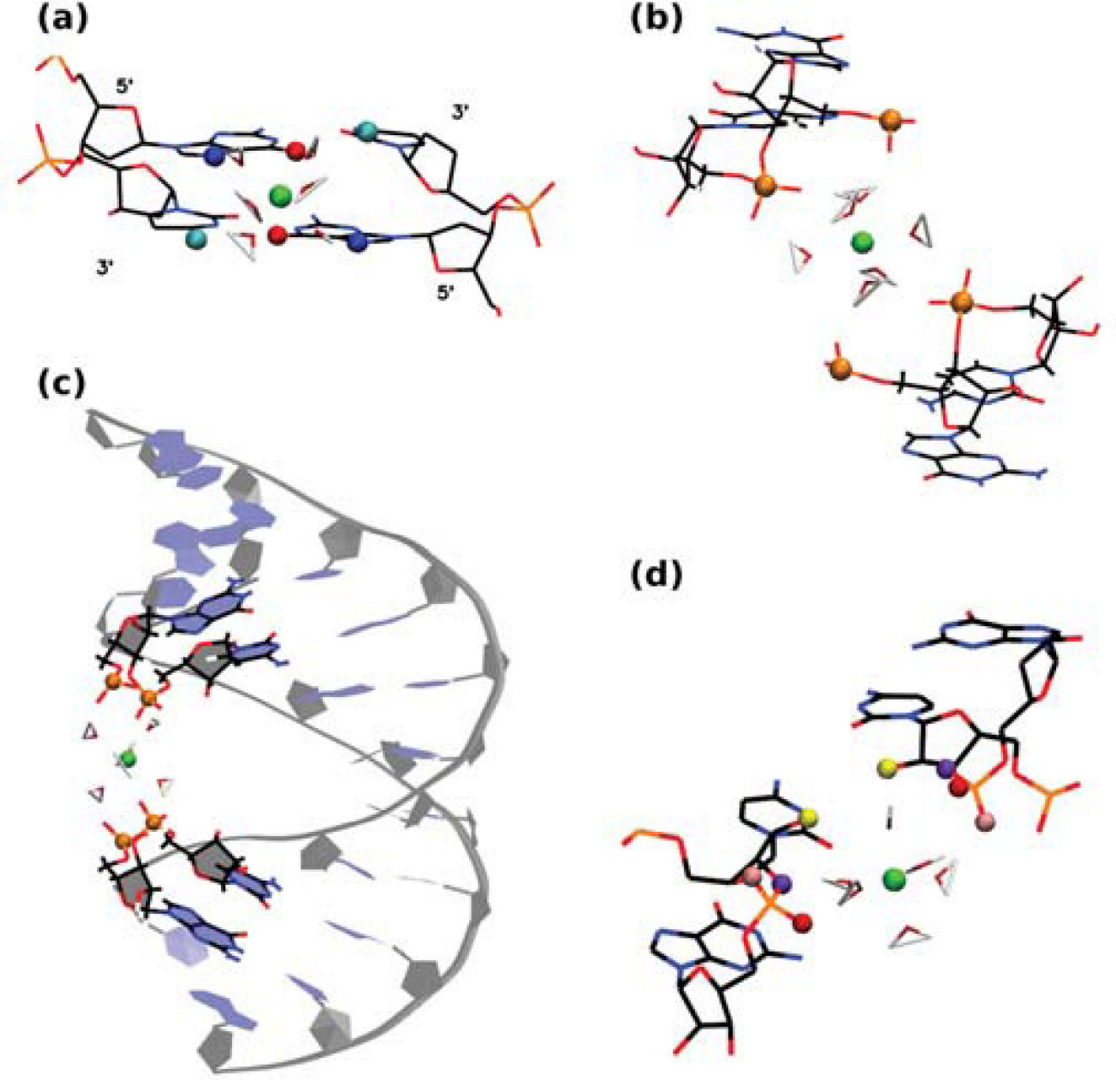
Atomistic details of binding sites of hexahydrated Mg^2+^ (green) for different nucleic acid structures. (**a**) Binding to G-O6 (red) and G-N7 (blue) (C-N4 is shown in cyan) in B-DNA; (**b**) binding to phosphate oxygens (orange) in A-RNA; (**c**) overall view of (b) along the duplex; (**d**) binding to G-OP1 (red), G-OP2 (pink), C-O2′ (yellow) and C-O3′ (violet) in Z-RNA in a similar arrangement as that shown in Figure 14c. The configurations shown are just a snapshot at a given time obtained from the MD simulations.

Table 3 also gives the characteristic residence times for the hexahydrated Mg[(H_2_O)_6_]^2+^ ions. Figure 15a shows Mg[(H_2_O)_6_]^2+^ localization in the major groove of B-DNA, with the hexahydrated ion bound to G-O6 (with a very large residence time of 9.4 ns) and G-N7 (and also in proximity of C-N4) in the GpC steps. The same binding can be found in A-RNA (see Figure S9a), with residence times of 2.4 ns (O6) and 2.2 ns (N4). These two cases are quite similar to those described for Na^+^ in Figure 14a and b and Figure S8. In addition, the ion can display long-time binding to the phosphate oxygens in A-RNA with equivalent residence times (2.1–2.3 ns). This occurs, for instance, in the bridge between distant phosphate groups as shown in Figure 15b and c. The latter results in high bending of the duplex, and in this way the Mg[(H_2_O)_6_]^2+^ ion completely closes access of other ions to the middle of the duplex. Z-DNA shows strong localization at the phosphate oxygens, with residence times of 7 ns (G-OP1) and 5.6 ns (G-OP2). This can be seen in Figure S9b, where the OP oxygens are pointing outward, away from the core of the helix, and in Figure S9c, where the ion is in the minor groove of Z-DNA. Z-RNA displays several binding sites: G-OP1 (7.6 ns), G-OP2 (5 ns), C-O2 (1.9 ns), C-O2′ (6 ns) and C-O3′ (4.9 ns). Figure 15d shows binding in the minor groove of Z-RNA.

### Nucleic acid structure as a function of salt concentration

We have checked whether the structure of the duplexes changes as a function of salt concentration. We consider the case for Na^+^ ion with 0-M, 0.4-M and 4.0-M excess NaCl salt. All the structures show typical alternating, periodic features in the various parameters that reflect the regular, alternating sequence pattern. Once the left-handed structures are equilibrated, they show no measurable sensitivity (within the statistical errors) to the salt concentration. B-DNA shows no sensitivity up to 0.4-M NaCl. However, for 4.0 M, some localized changes at base pairs 7–9 can probably be attributed to the high salt concentration. In particular, the sugar pucker of base G8 in B-DNA changes from its predominantly C2′-endo conformation to C1′-endo and some C2′-exo (Figure S10a). This affects other parameters around this base, such as the step twist shown in Figure S10b and the glycosidic angle, that changes from −112° at 0 M and 0.4 M to −58° at 4.0 M, effectively switching from an *anti* conformation to a ‘syn’ conformation. These local changes may indicate the onset of an instability due to the high salt concentration. On the other hand, A-RNA is the structure that shows most sensitivity to salt concentration globally. This is quite apparent in helical and step parameters. Two such examples are given in Figure S10c and d. Although the figures shown in Figure S10 are only averaged over the last 10 ns of the simulations, they give a good idea of the general trends. Inclination, step roll, helical and step twist and x-displacement all increase with salt concentration while helical and step rise, and propeller decrease with salt concentration. In other words, the structure becomes more compact and more A-like, an observation that has been reported before (85,87). For instance, we measure the average helical inclination and roll step parameter (during the last 10 ns) as ≃ 10° and ≃ 6°, respectively, for zero excess salt, and ∼17° and ≃ 10°, respectively, for 4.0-M NaCl.

## DISCUSSION AND CONCLUSIONS

### Role of the ion type

With MD simulations, we have calculated the distribution of the K^+^, Na^+^ and Mg^2+^ cations (and the Cl^−^ anions) around right-handed B-DNA and A-RNA, and left-handed Z-DNA and Z-RNA. As previously pointed out (76), differences in binding between the Na^+^ and K^+^ cations are important because while K^+^ cations dominate in the intracellular fluids (at ∼0.14 M), most studies have been carried out with the Na^+^ cation, which is mainly present in extracellular fluids. In fact, K^+^ cations are important for activating RNA systems, and for ribosome structure that can otherwise unfold in their absence (76,95,96). In solution, positive cations attract water molecules that form a ‘solvation shell’ around them. Large angle X-ray scattering and double difference infrared spectroscopy have determined (97) the bond distance between the cation and the first-shell water oxygen as 2.43 Å for Na^+^ and 2.81 Å for K^+^. This bond distance in Mg^2+^ is ∼2.00–2.15 Å (98). For the same average distance, we measure 2.44 Å for Na^+^, 2.89 Å for K^+^ and 2.00 Å for Mg^2+^, which provides for a good validation of the ion parameters being used.

The cations are naturally attracted to the electronegative sites around the nucleic acid duplexes, and both direct and water-mediated binding are seen. Many of the results can be understood by the fact that the ionic radius of each K^+^, Na^+^ and Mg^2+^ decreases in that order, while the strength of the first solvation shell around a metal cation decreases with its radius and increases with its charge. Thus, Mg^2+^ maintains its first solvation shell during the entire length of the simulations, and effectively acts as a hexahydrated cation, Mg[(H_2_O)_6_]^2+^. On the other hand, both monovalent cations can become partially dehydrated, but K^+^ (having a larger radius than Na^+^) can shed some of its first solvation waters with more ease and therefore penetrate deeper into smaller nucleic acid pockets. In fact, the hydration enthalpy of K^+^ has been measured experimentally to be ∼17 kcal/mol higher than that of Na^+^ (99,100). The Na^+^ ion can crystallize with full hydration shell with large low-symmetry counterions, while the inability of K^+^ to form well-defined hydrated structures in the solid state is a sign of its weak hydration (97).

Divalent ions are known to play an important role in the folding of biomolecules, especially RNA systems, and MD simulations have contributed to elucidate the role of cations in oligonucleotide systems. (35,57,101–107,102–107). In particular, Mg^2+^ cations are the most important divalent ions for the formation of RNA structures and their functional role in the cell. The fact that Mg^2+^ is very resistant to dehydration is reflected in crystallography, where Mg^2+^ in high-resolution crystal structures were observed to be fully hydrated (40,49). It has been seen that in crystallography, where concentrations are higher than physiological levels, Mg[(H_2_O)_6_]^2+^ can bind to sites that they would not normally do under physiological conditions, where they would wander off and be replaced by partially dehydrated monovalent cations (76). From the point of view of the simulations, it is known that accurate descriptions of divalent ions such as Mg^2+^ require the use of polarizable force fields (108), and the first simulations of DNA in explicit solvent with a fully polarizable force field (109–111) (applied to all atoms) showed that even a very simple representation of the atomic polarizability can improve the accuracy of the simulations. In spite of this, the parameters used in the present simulations (see SI) are known to give good qualitative insights into ion binding sites.

We have also looked at the distribution of the Cl^−^ anions and found that there were short-lived contacts, including contacts close to C-N4 in all four duplexes, to the O2′ sugar groups for the RNA duplexes, and to G-N2 in B-DNA and A-RNA, which confirms that it is possible for the anion to intrude upon the first hydration shell of nucleic acids (39). However, these events are quite rare and are not statistically significant. The CDFs (Figure 3) show that Cl^−^ anions generally are not allowed near the duplexes, particularly in the RNA structures. It is, however, possible that our simulations underestimate the contacts due to the absence of polarization. Studies on ion interface solvation (112–117) show that halide anions (all but fluoride) prefer surface solvation versus bulk solvation, and thus—in absence of other competing forces, such as those due to the presence of other ions—the halide ion would naturally migrate, for instance, to a biomolecule–water interface. The driving force for surface solvation is the polarizability of water. Thus, the inclusion of polarization in the force field could result in larger probabilities for closer contacts between co-ions and nucleic acids.

We have also tracked the diffusion constants for the different ions in the presence of different duplexes, and the results are presented in the SI.

### Comparison between different ions for B-DNA and A-RNA

Results for our sequence, (5′-CGCGCGCGCGCG-3′)_2_, show both similarities to and differences from other recent extensive simulations of ion distributions (74,73,78,86); the differences mainly due to the fact that the sequences employed in these other simulations are relatively rich in A and T or U nucleotides.

At zero excess salt, most Na^+^ direct binding to B-DNA occurs in the major groove, especially in the three central Gs in the GpC steps. For A-RNA, the Na^+^ direct binding is spread more evenly among all the Gs in the GpC steps of the major groove and is comparable to the binding to phosphate oxygens in the backbone. Direct binding to the major groove is saturated so that when the excess salt content increases to 0.4 M, direct binding to the major groove barely increases (direct binding to the phosphates increases slightly). For both duplexes and both salt concentrations, direct binding of Na^+^ in the minor groove is negligible. By contrast, more favorable binding of Na^+^ in minor grooves has been found in A-tract DNA or ApT steps both in NMR studies (61,62) and simulations (73,78). Naturally, the population of water-mediated Na^+^ contacts at 6 Å is much larger for each nucleotide than that for direct binding. Still, the occupation of the major groove in these water-mediated contacts is larger than the occupation of the minor groove, especially for A-RNA. For K^+^, direct binding is qualitatively similar to that of Na^+^, except that population in the major groove associated with the Gs in GpC steps is considerably increased, and there is a non-negligible population in the minor groove for B-DNA. On the other hand, minor groove occupation is almost non-existent for A-RNA (Figure 13). Preferred binding of K^+^ in the major groove has been observed experimentally (47,63).

Other general differences between B-DNA and A-RNA can be observed in the CDFs and RDFs presented in Figures 2,3,5–8. In B-DNA water-mediated binding of cations is more evenly distributed: while the majority resides in the major groove with occupancy varying between 40 and 56% for Na^+^ (Figure 12), the minor groove and backbone oxygens have non-negligible, comparable occupations around 20%. In A-RNA even water-mediated cations are more localized: occupancy in the major groove reaches up to 70%, it is very small for the minor groove, and rises again for the phosphate oxygens (Figures 12 and 13). Thus A-RNA exhibits a stronger localization of counterions, which is reflected in the higher peaks and faster decay in the CDFs in Figures 2 and 3. In A-RNA most of the cation density lies in the major groove along the cylindrical axis of the helix, a consequence of the structure of the helix whose base-pair planes are not perpendicular to the helix axis (as in B-DNA) but tilted toward the axis, thus leaving the core of the helix more exposed. This penetration of the ions into the open core of the helix is ‘complete’ for K^+^ at high salt (Figure 3), but even Mg[(H_2_O)_6_]^2+^ is very close to the helix axis (here calculated as the global z-axis with 3DNA, as in SCHNAaP (118), defined by vectors that are a combination of C1′ and G-N9/C-N4 atoms along the same strand, as developed by Rosenberg *et al.* (119)). Interestingly, a comparison with the Na^+^ CDFs for A-RNA for a different sequence with high content of A and U nucleotides (86) shows important sequence-dependent effects. For instance, the CDF for Na^+^ at 0.4-M NaCl in Figure 3, (86), shows several ‘binding regions’ with the maximum peak at ∼10 Å. For our CG sequence, the peak near the origin completely dominates the distribution of Na^+^ (an effect that is even larger for K^+^). This can be understood in terms of the perfect regularity of the pure CG sequence where the GpC steps strongly encourage binding to the major groove.

Particularly noticeable in our systems is the direct binding of the monovalent ions by G-O6 in the GpC steps, which has been observed before (70,74). A K^+^ ‘ion bridge’ of the monovalent cation joining the two electronegative G-O6 on different strands (Figure 14a,b) was observed both in DNA and RNA (70). In our results, this ion bridge also extends to the C-N4 atom in the corresponding GpC steps for both Na^+^ and K^+^, and for both B-DNA and A-RNA. Binding to C-N7 is also important (Figures 12 and 13), but with shorter residence times. This can be explained by the geometry of the steps. A cation between G-O6 and C-N7 will also be attracted by the backbone oxygens, and therefore its motion will be more diffusive, less localized. The absence of binding to the CpG steps was attributed to the protruding of the two electropositive cytosine amino groups in the CpG steps (70), which is consistent with our observations. Finally, with respect to the backbone oxygens, there is direct binding to O3′ and water-mediated binding to both O3′ and O5′. Naturally, A-RNA also shows binding to O2′, although this is not as strong as for O3′ and O5′ (Figure 7). Both phosphate oxygens have comparable binding in both forms, B-DNA and A-RNA, and for both Na^+^ and K^+^ (Figures 9 and 13). Residence times are considerably longer for K^+^ than for Na^+^. Long residence times near the phosphates have been also observed in some recent B-DNA simulations (74).

The Mg^2+^ cation has quite different binding properties from its monovalent counterparts, due to its tightly bound first solvation shell. In the CDFs at 0.2-M excess salt concentration in Figure 3, we observe two clearly defined binding regions for both B-DNA and A-RNA (and a very small intermediate peak for A-RNA). In agreement with the RDFs and CDFs, Figure 4 shows that the distribution of Mg^2+^ cations is more localized than those for Na^+^/K^+^ for all four duplexes. This strong localization of Mg^2+^ ions around A-RNA was observed recently (86), where it occurs not only in the presence of MgCl_2_ but also in mixtures with NaCl salt. The RDFs in Figure 6 show strong Mg[(H_2_O)_6_]^2+^ binding in the major groove to G-O6 at 4.1 Å (B-DNA) and 4.3 Å (A-RNA), with a much higher peak in B-DNA, and a second important peak further away for C-N4. An example of this binding geometry is given in Figure 15 a and Figure S8, where the residence times for G-O6 are 9.4 ns for B-DNA and 2.4 ns for A-RNA. The closest backbone oxygen bindings occur at O3′ and O5′ with a more distant peak for O2′ in A-RNA. Binding to the two phosphate oxygens is very strong, with the first peak strongly centered at 4.1 Å. An interesting case occurs when the hexahydrated ion binds phosphate pairs belonging to distant base pairs as shown for A-RNA in Figure 15b and c resulting in acute bending of the duplex and the prevention of access of other ions to the middle of the duplex. Binding to the minor groove is negligible, considerably less than that of Na^+^ or K^+^ at 6 Å. Binding of Mg[(H_2_O)_6_]^2+^ to the guanines in the major grooves has been measured previously (86) and has been seen experimentally (45,50,120,121).

With respect to the dependence of the right-handed forms on the salt concentration, we found that B-DNA is less sensitive than A-RNA. There are no structural changes in B-DNA at 0.4-M NaCl and only at the high concentration of 4.0-M NaCl excess salt, we see what can be interpreted as the start of a salt-induced instability in base G8. This is manifested as a flipping of G8 from the ‘anti’ to the ‘syn’ conformation. By contrast, A-RNA is quite sensitive to salt concentration, exhibiting global changes even at 0.4-M NaCl. The nature of these changes is in complete agreement with previous findings (85,87). Mainly, the structure becomes more compact and switches more to the A-form as salt concentration increases. This is seen in an increase of inclination, step roll, helical and step twist, and x-displacement with salt concentration, accompanied by a decrease of helical and step rise, and propeller. These trends are coupled, as an increase in helical inclination leads to a larger base pair roll (which follows the same pattern as Figure S9d), narrowing of the major groove and to a reduction of helical rise. It is not clear whether this is an effect of the force field (which at present is the most accurate one for the description of nucleic acids (87)), but it has been observed (87) that a different water model (SPC/E) can result in an even more compact A-RNA structure. Unfortunately, the existing experimental data includes a wide span of compactness for A-RNA duplexes, which precludes determining whether this trend toward compactness with increasing NaCl salt is real or not. The trend would seem counter-intuitive as a high increase in salt is expected to lead to a change in handedness, and thus one would expect a decrease of both helical and step twist, an increase of rise, etc. with salt concentration. Experimentally, however, the transition has been achieved under different conditions, such as chemical modification of the bases (122); high pressure (30) or different salt conditions, such as 6.0-M NaClO_4_ (29).

### Comparison for different ion distributions between the left-handed and the right-handed forms

There are similarities but also strong differences in ion distributions around the left-handed forms. These differences are measured not only when comparing to the right-handed forms but also when comparing between Z-DNA and Z-RNA.

Since ion distribution is a function of the duplex structure, we briefly review Z-DNA and Z-RNA structures. Both duplexes, formed by antiparallel strands with WC base-pairing, are characterized by the typical zig-zag pattern of the sugar-phosphate backbone, and a dinucleotide repeat unit. In Z-DNA the major and minor grooves are very similar in width, while in Z-RNA the base pairs are closer to the helix axis (with smaller *x*- and *y*-displacements) and both a deep, narrow minor groove and the major groove are well-defined. In addition to handedness, other major differences with the right-handed forms include the glycosyl angle: it is ‘anti’ for both G and C in the right-handed duplexes, but in the left-handed forms it is ‘syn’ for G and ‘anti’ for C. Both handedness and glycosyl angles have been used as highly discriminating order parameters for the description of the structural transition between B-DNA and Z-DNA, leading to a complex free energy landscape which allows for the coexistence of several competing mechanisms (24). The predominant sugar pucker (C2′-endo in B-DNA; C3′-endo in A-RNA) is C2′-endo for C and C3′-endo for G in the Z forms. Twist angles of 33° to 38° for B-DNA and 29° to 34° for A-RNA also change drastically. The Z forms have higher negative values for the GpC steps, and smaller values for the CpG steps. Both forms have intra-strand stacking for GpC steps and inter-strand stacking for CpG steps, as observed experimentally in Z-DNA (1), and in Z-RNA at low ionic strengths (31). In Z-RNA, the C-O2′ groups are deeply buried in the narrow minor groove, while the G-O2′ and the phosphate oxygens reside on the outer helix surface. We have maintained the atomic definition of major and minor grooves (as shown in Figure 1) to follow the convention in the literature for Z-DNA (1) and for Z-RNA (29), although from a geometrical point of view, the role of these grooves appears to be inverted for the left-handed forms.

CDFs in Figure 2,3 show that: (i) the ion distributions converge faster in the left-handed forms than in the right-handed forms; (ii) the maximum peaks are closer to the helical axis in the right-handed forms than in the left-handed forms; (iii) the relative height of the maximum peaks depends on the cation type. For Na^+^, first and second binding shells (when present) are higher in the Z forms than in the right-handed forms and, for each handedness, first peaks are higher for the RNA duplexes than for the DNA duplexes. While the K^+^ first peaks are considerably higher than those for the other two cations in the right-handed forms, they are equal to or lower than those of Na^+^ in the left-handed forms. Figure 4 shows that for any given distance before the asymptotic value, A-RNA is more screened than B-DNA. For regions close to the axis, A-RNA localizes more Na^+^ and K^+^ ions than both B-DNA and Z-DNA, and more Mg^2+^ ions than the three other forms. At intermediate distances, charge neutralization works better in the left-handed forms (which are comparable). For all duplexes, the Mg^2+^ ion distribution is considerably more localized than the Na^+^ ion distribution, which in turn is slightly more localized than the K^+^ ion distribution.

Binding to major and minor grooves changes dramatically when handedness changes (the role of the minor groove in the Z forms being closer to the role of the major groove in the right-handed forms). RDFs in Figure 5–9 show that (i) Na^+^ binding in the major groove for both B-DNA and A-RNA is similar, driven mainly by binding to G-O6, with localization also close to C-N4, followed by C-N7 in the GpC steps. (ii) Na^+^ binding in the minor groove of Z-DNA and Z-RNA is very different. Z-RNA has a very large peak for O2 at 2.3 Å and a second peak for N2 at 3.7 Å. Z-DNA has almost no binding at these short distances, with the first non-negligible peak for O2 at 4.6 Å. (iii) While Mg^2+^ binding in the major groove is stronger for B-DNA than A-RNA, its binding in the minor groove of Z-RNA is stronger than in Z-DNA. Binding to C-O2 shows two clearly defined bindings shells at 4.4 and 6.5 Å, which correspond to a direct binding to Mg[(H_2_O)_6_]^2+^ and binding with one intermediate water molecule to Mg[(H_2_O)_6_]^2+^. (iv) The O2′ in RNA naturally provides a source of binding differences between DNA and RNA. For Na^+^ the patterns of binding to O3′ and O5′ (and to less extent O4′) are relatively similar between B-DNA and A-RNA, with binding to O2′ less important than to O3′ and O5′. Instead, an important binding peak to O3′ at 2.5 Å in Z-DNA becomes minor in Z-RNA, while binding to O2′ at the same distance dominates. Similarly, for Mg^2+^ the patterns of binding to the O′ oxygens are similar between B-DNA and A-RNA, except for O2′ in A-RNA, which is not dominant. For Mg^2+^ in Z-RNA, the first binding peak to O3′ increases in Z-RNA with respect to Z-DNA, and the O2′ peak at 4.2 Å is also very important. (v) Binding of Na^+^ to the OP2 oxygens is similar for the four duplexes, while binding to OP1 increases for the Z forms. For Mg^2+^ , these bindings are comparable for the four duplexes.

Figure 10–13 show sequence-specific features. Both for zero and high salt concentration, Na^+^ direct binding to the major groove is small (K^+^ exhibits slightly more binding in the major groove) for both Z-DNA and Z-RNA. Indirect binding to the major groove increases the population (centered at the G's). The left-handed forms have distinct patterns of strong direct binding to the phosphate oxygens in the Gs (larger in Z-DNA than Z-RNA) and to the O′ oxygens in the Cs (larger in Z-RNA due to binding to O2′). With respect to the minor groove, direct binding of Na^+^ and K^+^ is quite different for Z-DNA and Z-RNA: in Z-DNA the ions do not bind directly to the minor groove, but they do so, with large occupation numbers, through intermediate waters; in Z-RNA direct binding to the minor groove is quite high, mainly due to C-O2. Mg^2+^ binding at 6 Å follows a similar pattern: not much binding in the major groove, with slightly more binding in the minor groove (but certainly not as high as for Na^+^ at 6 Å), preferential binding to the phosphate oxygens in the Gs, and to the O′ oxygens in the Cs.

In Z-DNA, the longest residence times for monovalent ions occur for G-OP1 (2.3 ns for Na^+^ and 4.7 ns for K^+^) with a second longest residence time at C-O3′. Thus, in the 5′-3′ direction the ion binds to the O3′ of the sugar ring of a cytosine and to the two phosphates G-OP1 and G-OP2 immediately following C-O3′. This, with the equivalent set of atoms one CpG step ahead in the opposite strand, forms a pocket, as illustrated in Figure 14 c for Na^+^ in Z-DNA. Table 2 indicates that for Na^+^, G-OP1 is a site of direct binding while G-OP2 and C-O3′ are sites of indirect binding. On the other hand, K^+^ finds itself in a tighter pocket, with direct binding to both G-OP1 and C-O3′, and indirect binding to G-OP2, which explains the longer residence times for K^+^ associated with these positions. Z-RNA seems to provide the best ‘ion trap’ with direct binding to the C-O2 and the C-O2′ atoms in a GpC step in one strand and the same set of atoms belonging to the same GpC step in the opposite strand (see example in Figure 14d). Bound to four RNA atoms, the ion only retains two of its first solvation shell waters. This ion bridge has been observed experimentally at physiological ionic strengths (31). These Z-RNA atoms exhibit the largest residence times for monovalent ions observed in our simulations: 7.8 ns (2.8 ns) for Na^+^ (K^+^) in C-O2 and 8.6 ns (5.4 ns) for Na^+^ (K^+^) in C-O2′. Finally, the hexahydrated Mg[(H_2_O)_6_]^2+^ ion also finds strong binding pockets in both Z-DNA and Z-RNA, specially when the phosphate oxygens are involved. Typical binding pockets are shown in Figure 15d and Figure S9b and c. Long residence times in Z-DNA include 7 ns for G-OP1 and 5.6 ns for G-OP2. Long residence times in Z-RNA include 6.0 ns in C-O2′, 4.9 ns in C-O3′, 7.6 ns in G-OP1 and 5.0 ns in G-OP2.

## SUPPLEMENTARY DATA

Supplementary Data are available at NAR Online.

SUPPLEMENTARY DATA

## References

[1] Wang A.H., Quigley G.J., Kolpak F.J., Crawford J.L., van Boom J.H., van der Marel G., Rich A. (1979). Molecular structure of a left-handed double helical DNA fragment at atomic resolution. Nature.

[2] Nordheim A., Rich A. (1983). The sequence (*dC* − *dA*)_*n*_.(*dG* − *dT*)_*n*_ forms left-handed Z-DNA in negatively supercoiled plasmids. Proc. Natl Acad. Sci. U.S.A..

[3] Rich A., Nordheim A., Wang A.H. (1984). The chemistry and biology of left-handed Z-DNA. Ann. Rev. Phys. Chem..

[4] Schroth G., Chou P., Ho P.J. (1992). Mapping Z-DNA in the human genome–computer-aided mapping revelas a nonrandom distribution of potential Z-DNA forming sequences in human genes. J. Biol. Chem..

[5] Liu L.F., Wang J.C. (1987). Supercoiling of the DNA template during transcription. Proc. Natl Acad. Sci. U.S.A..

[6] Oh D., Kim Y., Rich A. (2002). Z-DNA binding proteins can act as potent effectors of gene expression in vivo. Proc. Natl Acad. Sci. U.S.A..

[7] Lipps H.J. (1983). Antibodies against Z-DNA react with the macronucleus but not the micronucleus of the hypotrichous ciliate stylonychia mytilus. Cell.

[8] Lancillotti F., Lopez M., Alonso C., Stollar B. (1985). Locations of Z-DNA in polytene chromosomes. J. Cell Biol..

[9] Herbert A., Rich A. (1999). Left-handed Z-DNA: structure and function. Genetica.

[10] Kmiec E., Angelides K., Holloman W. (1985). Left-handed DNA and the synaptic pairing reaction promoted by ustilago rec1 protein. Cell.

[11] Blaho J., Wells R. (1987). Left-handed Z-DNA binding by the reca protein of escherichia-coli. J. Biol. Chem..

[12] Jaworski A., Hsieh W., Blaho J., Larson J., Wells R. (1987). Left-handed DNA in vivo. Science.

[13] Schwartz T., Rould M., Lowenhaupt K., Herbert A., Rich A. (1999). Crystal structure of the Z alpha domain of the human editing enzyme ADAR1 bound to left-handed Z-DNA. Science.

[14] Vinogradov A. (2003). DNA helix: the importance of being GC-rich. Nucleic Acids Res..

[15] Champ P., Maurice S., Vargason J., Champ T., Ho P. (2004). Distributions of Z-DNA and nuclear factor I in human chromosome 22; a model for coupled transcriptional regulation. Nucleic Acids Res..

[16] Wang G., Christensen L., Vasquez K. (2006). Z-DNA-forming sequences generate large-scale deletions in mammalian cells. Proc. Natl Acad. Sci. U.S.A..

[17] Fuertes M.A., Cepeda V., Alonso C., Pérez J.M. (2006). Molecular mechanisms for the B-Z transition in the example of poly[d(G-C)·d(G-C)] polymers. A critical review. Chem. Rev..

[18] Harvey S. (1983). DNA structural dynamics—longitudinal breathing as a possible mechanism for the B-reversible-Z transition. Nucleic Acids Res..

[19] Goto S. (1984). Characterization of intermediate conformational states in the B↔Z transition of *poly*(*dG* − *dC*) · *poly*(*dG* − *dC*). Biopolymers.

[20] Saenger W., Hienemann U. (1989). Raison d'etre and structural model for the B-Z transition of poly d(G-C)⋆poly d(G-C). FEBS Lett..

[21] Ansevin A., Wang A. (1990). Evidence for a new Z-type left-handed DNA helix - properties of Z(WC)-DNA. Nucleic Acids Res..

[22] Lim W., Feng Y. (2005). The stretched intermediate model of B-Z DNA transition. Biophys. J..

[23] Ha S.C., Lowenhaupt K., Rich A., Kim Y.G., Kim K.K. (2005). Crystal structure of a junction between B-DNA and Z-DNA reveals two extruded bases. Nature.

[24] Moradi M., Babin V., Roland C., Sagui C. (2012). Reaction path ensemble of the B-Z-DNA transition: a comprehensive atomistic study. Nucleic Acids Res..

[25] Hall K., Cruz P., Tinoco I., Jovin T., van de Sande J. (1984). Z-RNA—a left-handed RNA double helix. Nature.

[26] Adamiak R.W., Galat A., Skalski B. (1985). Salt- and solvent-dependent conformational transitions of ribo-CGCGCG duplex. Biochim. Biophys. Acta.

[27] Tinoco I. Jr., Cruz P., Davis P., Hall K., Hardin C.C., Mathies R.A., Puglisi J.D., Trulson M.A., Johnson W.C., Neilson T., van Knippenberg PH, Hilbers CW (1986). Z-RNA: a left-handed double helix.

[28] Popenda M., Biala E., Milecki J., Adamiak R.W. (1997). Solution structure of RNA duplexes containing alternating CG base pairs: NMR study of *r*(*CGCGCG*)_2_ and 2′ − *O* − *Me*(*CGCGCG*)_2_ under low salt conditions. Nucleic Acids Res..

[29] Popenda M., Milecki J., Adamiak R.W. (2004). High salt solution structure of a left-handed RNA double helix. Nucleic Acids Res..

[30] Krzyzaniak A., Barciszewski J., Furste J.P., Bald R., Erdmann V.A., Salanski P., Jurczak J. (1994). A-Z-RNA conformational changes effected by high pressure. Int. J. Biol. Macromol..

[31] Placido D., Brown B., Lowenhaupt K., Rich A., Athanasiadis A. (2007). A left-handed RNA double helix bound by the Z-alpha domain of the RNA-editing enzyme ADAR1. Structure.

[32] Rau D.C., Lee B., Parsegian V.A. (1984). Measurement of the repulsive force between polyelectrolyte molecules in ionic solution: hydration forces between parallel DNA double helices. Proc. Natl Acad. Sci. U.S.A..

[33] Knobler C., Gelbart W. (2009). Physical chemistry of DNA viruses. Annu. Rev. Phys. Chem..

[34] Fedor M. (2009). Comparative enzymology and structural biology of RNA self-cleavage. Annu. Rev. Biophys..

[35] Draper D.E., Grilley D., Soto A.M. (2005). Ions and RNA folding. Annu. Rev. Biophys. Biomol. Struct..

[36] Koculi E., Hyeon C., Thirumalai D., Woodson S.A. (2007). Charge density of divalent metal cations determines RNA stability. J. Am. Chem. Soc..

[37] Li P., Vieregg J., Tinoco I. (2008). How RNA unfolds and refolds. Annu. Rev. Biophys..

[38] MacKerrell A. Jr., Nilsson L. (2008). Molecular dynamics simulations of nucleic acid-protein complexes. Curr. Opin. Struct. Biol..

[39] Auffinger P., Bielecki L., Westhof E. (2004). Anion binding to nucleic acids. Structure.

[40] Prive G.G., Yanagi K., Dickerson R.E. (1991). Structure of the B-DNA decamer C-C-A-A-C-G-T-T-G-G and comparison with isomorphous decamers C-C-A-A-G-A-T-T-G-G and C-C-A-G-G-C-C-T-G-G. J. Mol. Biol..

[41] Shui X., Sines C.C., McFail-Isom L., Van Derveer D., Williams L.D. (1998). Structure of the potassium form of CGCGAATTCGCG: DNA deformation by electrostatic collapse around inorganic cations. Biochemistry.

[42] Shui X., McFail-Isom L., Hu G., Williams L.D. (1998). The B-DNA dodecamer at high resolution reveals a spine of water on sodium. Biochemistry.

[43] Tereshko V., Minasov G., Egli M. (1999). A ‘hydration’ spine in a B-DNA minor groove. J. Am. Chem. Soc..

[44] McFail-Isom L., Sines C.C., Williams L.D. (1999). DNA structure: cations in charge?. Curr. Opin. Struct. Biol..

[45] Ennifar E., Yusupov M., Walter P., Marquet R., Ehresmann B., Ehresmann C., Dumas P. (1999). The crystal structure of the dimerization initiation site of genomic HIV-1 RNA reveals an extended duplex with two adenine bulges. Struct. Fold. Des..

[46] Robinson H., Gao Y.G., Sanishvili R., Joachimiak A., Wang A. H.J. (2000). Hexahydrated magnesium ions bind in the deep major groove and at the outer mouth of A-form nucleic acid duplexes. Nucleic Acids Res..

[47] Howerton S.B., Sines C.C., VanDerveer D., Williams L.D. (2001). Locating monovalent cations in the grooves of B-DNA. Biochemistry.

[48] Subirana J.A., Soler-Lopez M. (2003). Cations as hydrogen bond donors: a view of electrostatic interactions in DNA. Annu. Rev. Biophys. Biomol. Struct..

[49] Chiu T.K., Dickerson R.E. (2000). 1 angstrom crystal structure of B-DNA real sequence specific and groove specific bending of DNA by magnesium and calcium. J. Mol. Biol..

[50] Ennifar E., Walter P., Dumas P. (2003). A crystallographic study of the binding of 13 metal ions to two related RNA duplexes. Nucleic Acids Res..

[51] Timsit Y., Bombard S. (2007). The 1.3 angstrom resolution structure of the RNA tridecamer r(GCGUUUGAAACGC): metal ion binding correlates with base unstacking and groove contraction. RNA.

[52] Tereshko V., Minasov G., Egli M. (1999). The Dickerson-Drew B-DNA dodecamer revisited at atomic resolution. J. Am. Chem. Soc..

[53] Chiu T.K., Kaczor-Grzeskowiak M., Dickerson R.E. (1999). Absence of minor groove monovalent cations in the crosslinked dodecamer CGCGAATTCGCG. J. Mol. Biol..

[54] Andresen K., Das R., Park H.Y., Smith H., Kwok L.W., Lamb J.S., Kirkland E.J., Herschlag D., Finkelstein K.D., Pollack L. (2004). Spatial distribution of competing ions around DNA in solution. Phys. Rev. Lett..

[55] Chu V., Bai Y., Lipfert J., Herschlag D., Doniach S. (2008). A repulsive field: advances in the electrostatics of the ion atmosphere. Curr. Opin. Chem. Biol..

[56] Wong G. C.L., Pollack L. (2010). Electrostatics of strongly charged biological polymers: ion-mediated interactions and self-organization in nucleic acids and proteins. Annu. Rev. Phys. Chem..

[57] Bai Y., Greenfeld M., Travers K., Chu V., Lipfert J., Doniach S., Herschlag D. (2007). Quantitative and comprehensive decomposition of the ion atmosphere around nucleic acids. J. Am. Chem. Soc..

[58] Hud N.V., Feigon J. (1997). Localization of divalent metal ions in the minor groove of DNA A-tracts. J. Am. Chem. Soc..

[59] Hud N.V., Sklenar V., Feigon J. (1999). Localization of ammonium ions in the minor groove of DNA duplexes in solution and the origin of DNA A-tract bending. J. Mol. Biol..

[60] Bonvin A. (2000). Localisation and dynamics of sodium counterions around DNA in solution from molecular dynamics simulation. Eur. Biophys. J..

[61] Denisov V.P., Halle B. (2000). Sequence-specific binding of counterions to B-DNA. Proc. Natl Acad. Sci. U.S.A..

[62] Marincola F.C., Denisov V.P., Halle B. (2004). Competitive Na+ and Rb+ binding in the minor groove of DNA. J. Am. Chem. Soc..

[63] Maehigashi T., Hsiao C., Woods K., Moulaei T., Hud N.V., Williams L.D. (2012). B-DNA structure is intrinsically polymorphic: even at the level of base pair positions. Nucleic Acids Res..

[64] Manning G.S. (1978). Molecular theory of polyelectrolyte solutions with applications to electrostatic properties of polynucleotides. Q. Rev. Biophys..

[65] Bacquet R.J., Rossky P.J. (1988). Ionic distributions and competitive association on DNA mixed salt solutions. J. Phys. Chem..

[66] York D.M., Darden T., Deerfield D., Pedersen L.G. (1992). The interaction of Na(I), Ca(II), and Mg(II) metal-ions with duplex DNA—a theoretical modeling study. Intl. J. Quant. Chem..

[67] Chen S.W.W., Honig B. (1997). Monovalent and divalent salt effects on electrostatic free energies defined by the nonlinear Poisson-Boltzmann equation: application to DNA binding reaction. J. Phys. Chem. B.

[68] Young M.A., Jayaram B., Beveridge D.L. (1997). Intrusion of counterions into the spine of hydration in the minor groove of B-DNA: fractional occupancy of electronegative pockets. J. Am. Chem. Soc..

[69] Feig M., Pettitt B.M. (1999). Sodium and chlorine ions as part of the DNA solvation shell. Biophys. J..

[70] Auffinger P., Westhof E. (2000). Water and ion binding around RNA and DNA (C,G) oligomers. J. Mol. Biol..

[71] Orozco M., Pérez A., Noy A., Luque F.J. (2003). Theoretical methods for the simulation of nucleic acids. Chem. Soc. Rev..

[72] Rueda M., Cubero E., Laughton C.A., Orozco M. (2004). Exploring the counterion atmosphere around DNA: what can be learned from molecular dynamics simulations?. Biophys. J..

[73] Ponomarev S.Y., Thayer K.M., Beveridge D.L. (2004). Ion motions in molecular dynamics simulations on DNA. Proc. Natl Acad. Sci. U.S.A..

[74] Várnai P., Zakrzewska K. (2004). DNA and its counterions: a molecular dynamics study. Nucleic Acids Res..

[75] Taubes C.H., Mohanty U., Chu S. (2005). Ion atmosphere around nucleic acid. J. Phys. Chem. B.

[76] Auffinger P., Hashem Y. (2007). Nucleic acid solvation: from outside to insight. Curr. Opin. Struct. Biol..

[77] Garcia A.E., Paschek D. (2008). Simulation of the pressure and temperature folding/unfolding equilibrium of a small RNA hairpin. J. Am. Chem. Soc..

[78] Yoo J., Aksimentiev A. (2012). Competitive binding of cations to duplex DNA revealed through molecular dynamics simulations. J. Phys. Chem. B.

[79] Perez A., Luque F.J., Orozco M. (2012). Frontiers in molecular dynamics simulations of DNA. Acc. Chem. Res..

[80] Cisneros G.A., Karttunen M., Ren P., Sagui C. (2014). Classical electrostatics for biomolecular simulations. Chem. Rev..

[81] York D.M., Darden T.A., Pedersen L.G. (1993). The effect of long-range electrostatic interactions in simulations of macromolecular crystals: a comparison of the Ewald and truncated list methods. J. Chem. Phys..

[82] Darden T.A., York D.M., Pedersen L.G. (1993). Particle mesh Ewald: an N log(N) method for Ewald sums in large systems. J. Chem. Phys..

[83] York D.M., Yang W., Lee H., Darden T.A., Pedersen L.G. (1995). Towards the accurate modeling of DNA: the importance of long-range electrostatics. J. Am. Chem. Soc..

[84] Essmann U., Perera L., Berkowitz M.L., Darden T., Lee H., Pedersen L.G. (1995). A smooth particle mesh Ewald method. J. Chem. Phys..

[85] Besseova I., Otyepka M., Reblova K., Sponer J. (2009). Dependence of A-RNA simulations on the choice of the force field and salt strength. Phys. Chem. Chem. Phys..

[86] Kirmizialtin S., Elber R. (2010). Computational exploration of mobile ion distributions around RNA duplex. J. Phys. Chem. B.

[87] Besseova I., Barnas P., Kuhrova P., Kosinova P., Otyepka M., Sponer J. (2012). Simulations of A-RNA duplexes. The effect of sequence, solute force field, water model, and salt concentration. J. Phys. Chem. B.

[88] Kirmizialtin S., Pabit S.A., Meisburger S.P., Pollack L., Elber R. (2012). RNA and its ionic cloud: solution scattering experiments and atomically detailed simulations. Biophys. J..

[89] Kirmizialtin S., Silalahi A.R., Elber R., Fenley M.O. (2012). The ionic atmosphere around A-RNA: Poisson-Boltzmann and molecular dynamics simulations. Biophys. J..

[90] Perez A., Marchan I., Svozil D., Sponer J., Cheatham T.E., Laughton C.A., Orozco M. (2007). Refinement of the AMBER force field for nucleic acids: improving the description of α/γ conformers. Biophys. J..

[91] Banas P., Hollas D., Zgarbva M., Jurecka P., Orozco M., Cheatham T.E., Sponer J., Otyepka M. (2010). Performance of molecular mechanics force fields for RNA simulations: stability of UUCG and GNRA hairpins. J. Chem. Theory Comput..

[92] Zgarbova M., Otyepka M., Sponer J., Mladek A., Banas P., Cheatham T.E., Jurecka P. (2011). Refinement of the Cornell et al. nucleic acids force field based on reference quantum chemical calculations of glycosidic torsion profiles. J. Chem. Theory Comput..

[93] Case D.A., Darden T.A., Cheatham T.E., Simmerling C.L., Wang J., Duke R.E., Luo R., Walker R.C., Zhang W., Merz K.M. (2012). AMBER 12.

[94] Jorgensen W.L., Chandrasekhar J., Madura J., Klein M.L. (1983). Comparison of simple potential functions for simulating liquid water. J. Chem. Phys..

[95] Naslund P.H., Hultin T. (1971). Structural and functional defects in mammalian ribosomes after potassium deficiency. Biochim. Biophys. Acta.

[96] Ennis H.L., Artman M. (1972). Ribosome size distribution in extracts of potassium-depleted Escherichia coli. Biochem. Biophys. Res. Commun..

[97] Mahler J., Persson I. (2012). A study of the hydration of the alkali metal ions in aqueous solution. Inorg. Chem..

[98] Ohtaki H., Radnai T. (1993). Structure and dynamics of hydrated ions. Chem. Rev..

[99] Tissandier M.D., Cowen K.A., Feng W.Y., Gundlach E., Cohen M.H., Earhart A.D., Coe J.V., Tuttle T.R. Jr. (1998). The proton's absolute aqueous enthalpy and Gibbs free energy of solvation from cluster-ion solvation data. J. Phys. Chem. A.

[100] Schmid R., Miah A.M., Sapunov V.N. (2000). A new table of the thermodynamic quantities of ionic hydration: values and some applications (enthalpy-entropy compensation and Born radii). Phys. Chem. Chem. Phys..

[101] Thirumalai D., Lee N., Woodson S.A., Klimov D.K. (2001). Early events in RNA folding. Annu. Rev. Phys. Chem..

[102] Auffinger P., Bielecki L., Westhof E. (2003). The Mg2+ binding sites of the 5S rRNA loop E motif as investigated by molecular dynamics simulations. Chem. Biol..

[103] Das R., Kwok L.W., Millett I.S., Bai Y., Mills T.T., Jacob J., Maskel G.S., Seifert S., Mochrie S. G.J., Thiyagarajan P. (2003). The fastest global events in RNA folding: electrostatic relaxation and tertiary collapse of the tetrahymena ribozyme. J. Mol. Biol..

[104] Auffinger P., Bielecki L., Westhof E. (2004). Symmetric K+ and Mg2+ ion-binding sites in the 5S rRNA loop E inferred from molecular dynamics simulations. J. Mol. Biol..

[105] Woodson S.A. (2005). Metal ions and RNA folding: a highly charged topic with a dynamic future. Curr. Opin. Chem. Biol..

[106] Das R., Travers K.J., Bai Y., Herschlag D. (2005). Determining the *Mg*^2+^ stoichiometry for folding an RNA metal ion core. J. Am. Chem. Soc..

[107] Grilley D., Soto A.M., Draper D.E. (2006). *Mg*^2+^-RNA interaction free energies and their relationship to the folding of RNA tertiary structures. Proc. Natl Acad. Sci. U.S.A..

[108] Jiao D., King C., Grossfield A., Darden T., Ren P. (2006). Simulation of Ca2+ and Mg2+ solvation using polarizable atomic multipole potential. J. Phys. Chem. B.

[109] Baucom J., Transue T., Fuentes-Cabrera M.A., Krahn J.M., Darden T., Sagui C. (2004). Molecular dynamics simulations of the d(CCAACGTTGG)_2_ decamer in crystal environment: comparison of atomic point-charge, extra-point and polarizable force fields. J. Chem. Phys..

[110] Babin V., Baucom J., Darden T.A., Sagui C. (2006). Molecular dynamics simulations of DNA with polarizable force fields: convergence of an ideal B-DNA structure to the crystallographic structure. J. Phys. Chem. B.

[111] Babin V., Baucom J., Darden T.A., Sagui C. (2006). Molecular dynamics simulations of polarizable DNA in crystal environment. Int. J. Quantum Chem..

[112] Perera L., Berkowitz M.L. (1991). Many-body effects in molecular-dynamics simulations of Na^+^(H_2_O)_*n*_ and Cl^−^(H_2_O)_*n*_ clusters. J. Chem. Phys..

[113] Markovich G., Giniger R., Levin M., Cheshnovsky O. (1991). Photoelectron-spectroscopy of iodine anion solvated in water clusters. J. Chem. Phys..

[114] Perera L., Berkowitz M.L. (1992). Structure and dynamics of *Cl*^−^(*H*_2_*O*)_20_ clusters: the efect of the polarizability and the charge of the ion. J. Chem. Phys..

[115] Perera L., Berkowitz M.L. (1993). Stabilization energies of Cl^−^, Br^−^, and I^−^ ions in water clusters. J. Chem. Phys..

[116] Perera L., Berkowitz M.L. (1993). Many-body effects in molecular-dynamics simulations of Na^+^(H_2_O)_*n*_ and Cl^−^(H_2_O)_*n*_ clusters (VOL 95, PG 1954, 1991). J. Chem. Phys..

[117] Herce D.H., Perera L., Darden T.A., Sagui C. (2004). Surface solvation for an ion in a water cluster. J. Chem. Phys..

[118] Lu X.J., Hassan M.A.E., Hunter C.A. (1997). Structure and conformation of helical nucleic acids: analysis program (SCHNAaP). J. Mol. Biol..

[119] Rosenberg J.M., Seeman N.C., Day R.O., Rich A. (1976). RNA double helices generated from crystal-structures of double helical dinucleoside phosphates. Biochem. Biophys. Res. Commun..

[120] Froystein N., Davis J., Reid B., Sletten E. (1993). Sequence-selective metal-ion binding to DNA oligonucleotides. Acta Chem. Scand..

[121] Cate J., Hanna R., Doudna J. (1997). A magnesium ion core at the heart of a ribozyme domain. Nat. Struct. Biol..

[122] Uesugi S., Ohkuko M., Urata H., Ikehara M., Kobayshi Y., Kyogoki Y. (1984). Ribooligonucleotides r(C-G-C-G) analogues containing 8-substituted guanosine residues form left-handed duplexes with Z-form-like structures. Am. Chem. Soc..

