# Supplementary material for: Ion distributions around left- and right-handed DNA and RNA duplexes: a comparative study
Source: Nucleic Acids Res. 2014 Nov 26;42(22):13981–96. doi: 10.1093/nar/gku1107 (PMC4267617; doi:10.1093/nar/gku1107)
Supplement: SUPPLEMENTARY DATA [file supp_42_22_13981__index.html]

Ion distributions around left- and right-handed DNA and RNA duplexes: a comparative study — Ion distributions around left- and right-handed DNA and RNA duplexes: a comparative study — SUPPLEMENTARY DATA 

# Ion distributions around left- and right-handed DNA and RNA duplexes: a comparative study

## SUPPLEMENTARY DATA

**Files in this Data Supplement:**

- SUPPLEMENTARY DATA
