# Supplementary material for: Ion distributions around left- and right-handed DNA and RNA duplexes: a comparative study
Source: Nucleic Acids Res. 2014 Nov 26;42(22):13981–96. doi: 10.1093/nar/gku1107 (PMC4267617; doi:10.1093/nar/gku1107)
Supplement: SUPPLEMENTARY DATA [file supp_gku1107_sup_2.pdf]

# Ion distributions around left- and right-handed DNA and RNA duplexes: a Molecular Dynamics study [Supplementary Information]

Feng Pan, Christopher Roland, Celeste Sagui*

Center for High Performance Simulations (CHiPS) and Department of Physics, North Carolina State University, Raleigh, NC 27695-8202.

∗ E-mail: sagui@ncsu.edu

## 1 Materials and Methods

Large-scale MD simulations were used to explore the ion distribution around DNA and RNA sequences $(CG)_6$ in an explicit solvent environment. The simulations were carried out using the PMEMD module of the AMBER 12 [1] software package with the ff12SB force field with parameters ff99BSC0 [2] for DNA and ff99BSC0+$\chi_{OL3}$ [3, 4] for RNA. The TIP3P model [5] was used for the water molecules. The ion parameters are relatively standard and offered in the AMBER force field employed. The monovalent ion parameters for Ewald and TIP3P waters are from Ref. [6], while the the $Mg^{2+}$ parameters were taken from from Aqvist's work [7].

**Table 1. Force field parameters for different ions**

| | radius(Å) | mass(u) | epsilon(kcal/mol) |
|---|---|---|---|
| $Na^+$ | 1.369 | 22.99 | 0.0874393 |
| $K^+$ | 1.705 | 39.10 | 0.1936829 |
| $Mg^{2+}$ | 0.7926 | 24.305 | 0.8947 |
| $Cl^-$ | 2.513 | 35.45 | 0.0355910 |

The long-range Coulomb interaction was evaluated by means of the Particle-Mesh Ewald (PME) method [8] with a 9 Å cutoff and an Ewald coefficient of 0.30768. Similarly, the van der Waals interaction were calculated by means of a 9 Å atom-based nonbonded list, with a continuous correction applied to the long-range part of the interaction. The production runs were generated using the leap-frog algorithm with a 1 fs timestep with Langevin dynamics with a collision frequency of 1 $ps^{-1}$. Data was saved every picosecond of the simulation. The SHAKE algorithm was applied to all bonds involving hydrogen atoms. The nucleic acid structures generated by means of these constant temperature ($T = 300K$) and pressure (1 atm) runs were analyzed by means of the 3DNA (v2.1) package [9] and the PTRAJ package

in AMBERTOOLS [1].

The initial coordinates of the nucleic acid structures were generated as follows. The usual right-handed B-DNA and A-RNA structures were generated using the NAB package in AMBERTOOLS (ver.12) in Arnott's conformation [10, 11], while the left-handed Z-DNA structure was available from our previous investigations [12]. For the Z-RNA structure, we first generated the $(CG)_3$ structure of Z-DNA, and then modified it to generate the corresponding RNA structure. The resulting structure was then equilibrated for 10 *ns* in an explicit solvent environment with a neutralizing number of $Na^+$ ions. The average structure was then analyzed using 3DNA and compared to the NMR-based Z-RNA structure found in the PDB data bank (PDB id: 1T4X) [13]. Since the local base-pair and step parameters of this equilibrated structure were found to be quite close to the experimental structure, we simply replicated the $(CG)_3$ structure to generate the initial $(CG)_6$ Z-RNA duplex. The duplexes were first equilibrated in a smaller water box (with neutralizing ions) and then placed in a larger cubic box of $\sim$ 82 Å side, filled with a suitable number of water molecules. Such a large box is necessary, since cylindral distribution functions need to be calculated to a distance of at least 30 Å. To achieve electroneutrality, 22 water molecules were replaced by $Na^+$ cations. For simulations involving high salt concentrations, the number of water molecules replaced by salt anions and cations was dictated by the system size and density. In order to minimize any correlation effects on the initial distribution of the ions, these were placed at random at a distance of 10 Å away from each other and the duplex structure.

Five simulations were carried out for each duplex with the following ions: (i) 22 neutralizing $Na^+$, no excess salt; (ii) 22 neutralizing $Na^+$, and 0.4 M NaCl; (iii) 22 neutralizing $K^+$, and 0.4 M KCl; (iv) 11 neutralizing $Mg^{2+}$ and 0.2 M $MgCl_2$; and (v) 22 neutralizing $Na^+$, and 4.0 M NaCl. Here, the "salt" molarity is used to indicate *excess* salt (over the neutralizing ions). Thus, we will refer to the case (i) above as "zero salt", although it has 0.06M $Na^+$, due to the neutralizing ions.

The energy of each final system of oligonucleotide, waters and ions was then minimized with the nucleic acid and ions fixed, followed by further minimization where atomic motion was allowed. Subsequently, the temperature was gradually raised at constant volume from zero to 300 K over a period of 50 ps, with the DNA/RNA structure and ions restrained with a harmonic constant of 50 kcal/mol. The resulting structure was then further equilibrated at 300 K for 100 ps. The restraints on the duplex atoms and ions were then gradually reduced by decreasing the restraining harmonic constant in 5 steps during equal intervals of time. The final configuration was then used for a 1 ns constant pressure simulation

which was completely unrestrained, thereby adjusting the box size so as to achieve a system density of approximately 1 g/ml. This was then followed by 120 ns of constant pressure and temperature production runs. Equilibrium data was collected from only the last 100 ns of these runs, since a minimum of 20 ns is required in order to stabilize the ion distribution.

Our analysis was focused on calculating the distribution of the mobile ions around the nucleic acid structures. To that end, we calculated the diffusion coefficients for the ions, the cylindrical and radial distribution functions, and carried out an analysis of the efficacy of different sites to localize the ions. The diffusion coefficient (D) for the ions was obtained by conventional means:

$$D = \left\langle |\vec{r}(t) - \vec{r}(0)|^2 \right\rangle / (6\Delta t), \tag{1}$$

*i.e.*, D is obtained by calculating the slope of the mean-square displacement as a function of time. We calculated this quantity over a 10 ns time interval, taking data every 10 ps, and averaging the result over all the ions. Turning to the radial distribution function (RDF), this is defined in terms of the distance $r$ of an atom B from a central atom A:

$$g_{AB}(r) = \frac{\rho_B(r \mid r_A = 0)}{\rho_B}, \tag{2}$$

where $\rho_B$ is the bulk density of B and $\rho_B(r \mid r_A = 0)$ is the conditional distance-dependent density of atoms of type B a distance $r$ from central site A. In practice the RDF is calculated as:

$$g_{AB}(r) = \left(\frac{V}{N_B}\right) \frac{N_B(r, \Delta)}{V_B(r, \Delta)} = \left(\frac{V}{N_B}\right) \frac{N_B(r, \Delta)}{4\pi r^2 \Delta} \tag{3}$$

where $N_B(r, \Delta)$ is the average number of atoms of type B located between distances $r - \frac{1}{2}\Delta$ and $r + \frac{1}{2}\Delta$ from central atom A, and $V_B(r, \Delta)$ is the volume of the spherical slice between $r - \frac{1}{2}\Delta$ and $r + \frac{1}{2}\Delta$. RDFs are particularly useful for investigating the binding properties between specific atoms such as negatively charged nitrogens and oxygens on the duplexes and positively charged ions. In these RDFs the location of the peaks reveals the typical binding distances. In the results presented here, the RDFs between two atom types were computed as an average over all the equivalent atom pairs along the duplex. An ion can be attracted to more than one atom in the duplex, and thus it can contribute to the RDFs of different atom types in the duplex. A particularly useful variant of RDFs involves the cylindrical distribution

functions, which track the ion concentration measured radially outwards from the central axis of the duplex. The calculation of these functions is the same as for the RDFs, except that $r$ is measured on the plane perpendicular to the duplex axis and $V_B(r, \Delta)$ now represents the volume of a cylindrical slice between $r-\Delta/2$ and $r+\Delta/2$ from the chosen central axis. In our analysis, the global z-axis was calculated by the package 3DNA, as in SCHNAaP [14]. The vectors used to define the axis are a combination of C1' and G-N9/C-N4 atoms along the same strand, as developed by Rosenberg *et al.* [15]. Calculating the cylindrical distribution function not only gives insight into the properties of the ion distribution, but also provides for a good check for the convergence of the simulation.

## 2 Results

**Diffusion Constants**. Table 2 summarizes the calculated diffusion coefficients for cations in the presence of the different duplexes. Generally speaking, these results are in good agreement with values obtained in B-DNA studies [16]. First, we consider the results for the $Na^+$ ion. At low ion concentration, the diffusion coefficients for the right-handed structures are higher than for the corresponding left-handed structures. For comparison, the calculated diffusion coefficient for 22 free $Na^+$ ions in TIP3P waters is $1.56 \times 10^{-9} m^2/s$, which is higher than the experimental value of $1.33 \times 10^{-9} m^2/s$ for ions in pure water [17]. The reduced values for Z-DNA/Z-RNA are indicative of a reduced mobility, due to more binding to the nucleic acids. At high salt concentrations (0.4 M), the diffusion coefficients increase for all structures, and their values are comparable to that of the free ion. Here, the number of mobile ions overwhelms the number of localized ions, giving rise to increased values of the diffusion coefficients. Table 2 also presents the diffusion coefficients for $K^+$ at 0.4 M KCl and for $Mg^{2+}$ at 0.2 M $MgCl_2$. As a percentage of their bulk values (computed in TIP3P waters) the $Mg^{2+}$ ion has the smallest value, indicating that it is in general more localized.

**Table 2. Diffusion coefficient of cations (in units of $10^{-9} m^2/s$) for the four different nucleic acid structures.** Coefficients are given for a duplex system without excess salt, and for the high excess salt cases. For comparison, the calculated diffusion coefficient in TIP3P waters is 1.56±0.14 for $Na^+$ ions, 2.59±0.14 for $K^+$ ions and 1.07±0.11 for $Mg^{2+}$ ions.

| Structure | $Na^+$ (no salt) | $Na^+$ (0.4M) | $K^+$ (0.4M) | $Mg^{2+}$ (0.2M) |
|---|---|---|---|---|
| B-DNA | 1.40±0.15 | 1.48±0.08 | 2.28±0.17 | 0.90±0.10 |
| Z-DNA | 0.98±0.13 | 1.44±0.03 | 2.16±0.11 | 0.89±0.09 |
| A-RNA | 1.28±0.19 | 1.45±0.10 | 2.26±0.13 | 0.92±0.10 |
| Z-RNA | 1.04±0.14 | 1.48±0.10 | 2.38±0.12 | 0.90±0.09 |

**Comparison with the solution of the linear Poisson-Boltzmann equation.** Over the years, a number of continuum-based theories have been proposed to model the concentration profile of ions away from charged biomolecules. Two particularly prominent examples include the so-called Manning condensation theory [18], and theories based on various versions of the Poisson-Boltzmann equation. At short distances near the duplexes, these theories are expected to fail because the atomic details matter [19]. However at larger distances, continuum theories can work. To probe this point, we considered a simple model consisting of two concentric cylinders (inner cylinder radius r=a; outer cylinder, r=b), with a constant charge density embedded on the inner cylinder and the electric field vanishing on the outer cylinder. The potential generated by such a model may be solved analytically using the linearized Poisson-Boltzmann equation [20]. The application of this model to our systems gives excellent results at larger distances. For example, SI Fig.11 shows results for 0.4 M $Na^+$ (radii a=12.0 Å and b=29.5 Å ) and shows that this simple continuum model well represents the cylindrical ion distribution for distances larger than about 12.0 Å (RNA) and 14.0 Å (DNA).

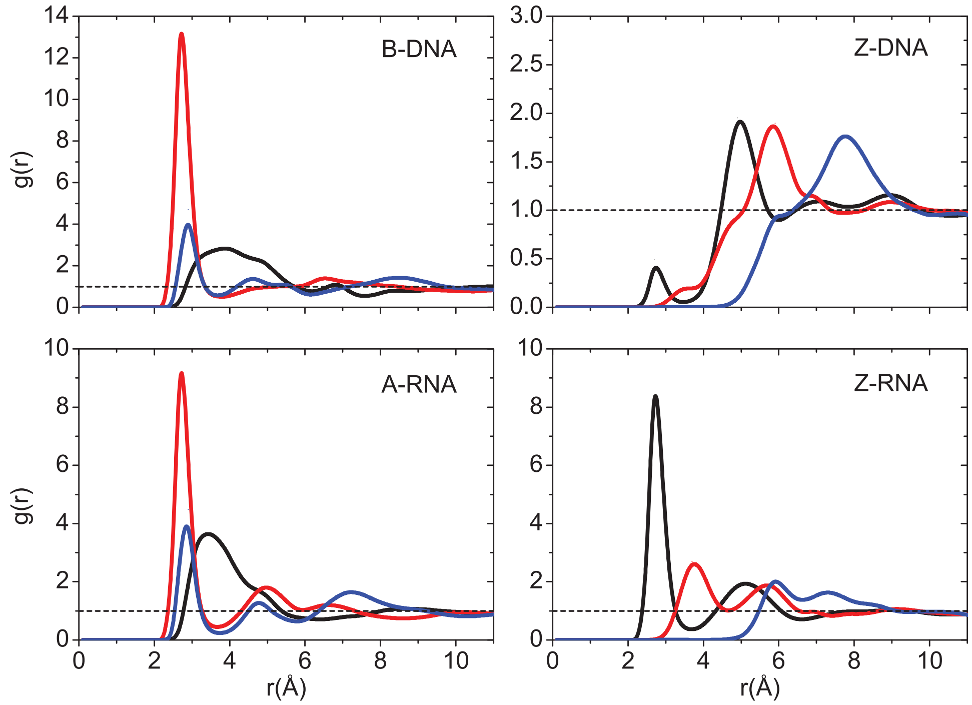


**Supplementary Figure 1. RDFs for $K^+$ with respect to major and minor groove atoms at 0.4 M salt concentration.** For B-DNA and A-RNA, the colors indicate RDFs with respect to atoms in the major groove: O6 (red), N7 (blue), N4 (black). For Z-DNA and Z-RNA, the colors indicate RDFs with respect to atoms in the minor groove: O2 (black), N2 (red) and N3 (blue).

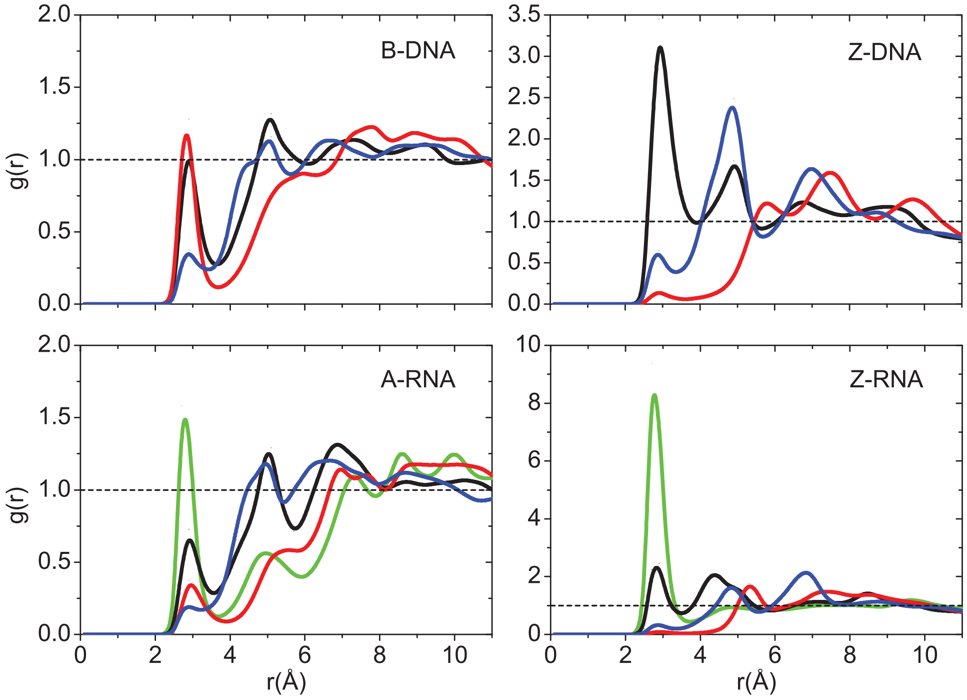


**Supplementary Figure 2. RDFs for $K^+$ with respect to O' backbone oxygens at 0.4 M salt concentration.** Colors indicate: O2' (green), O3' (black), O4' (red), and O5' (blue).

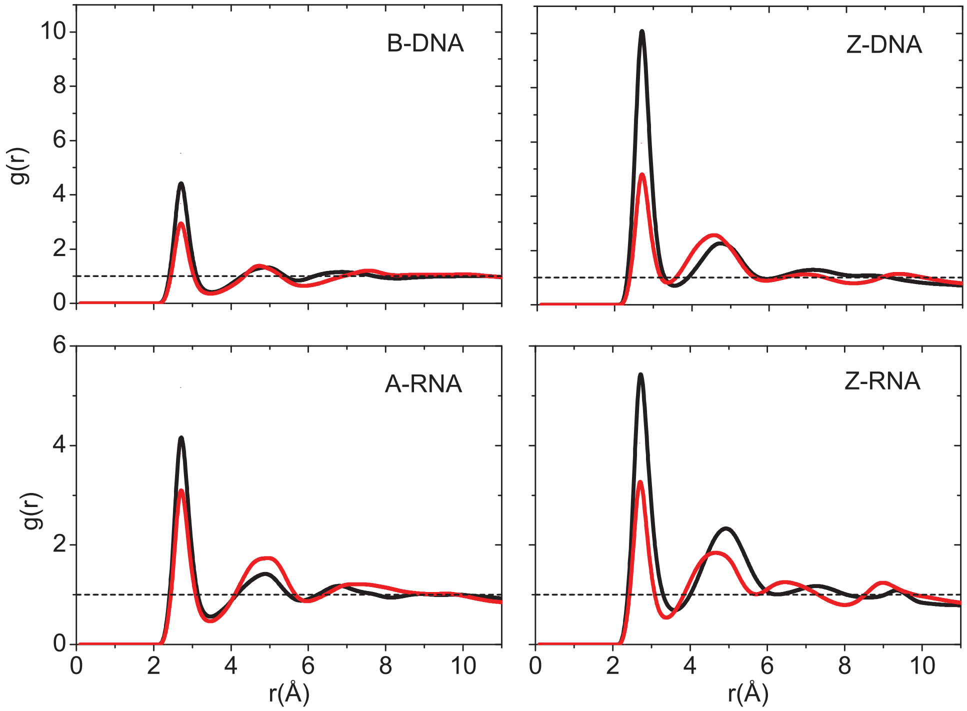


**Supplementary Figure 3. RDFs for $K^+$ with respect to phosphate oxygens at 0.4 M salt concentration.** Colors indicate: OP1 (black), OP2 (red). The maximum for the four duplexes occurs approximately at 2.8 Å.

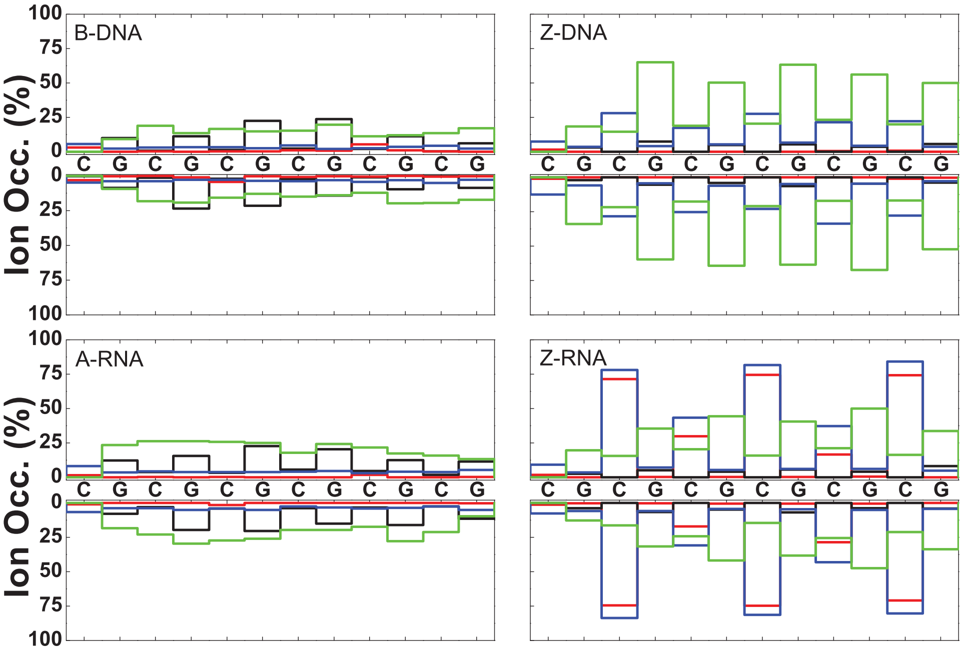


**Supplementary Figure 4. $Na^+$ ion occupancies within 3 Å as function of sequence for 0.4M salt concentration.** Colors represent: major groove (black), minor groove (red), O' oxygen atoms on backbone (blue), and phosphate oxygens (green).

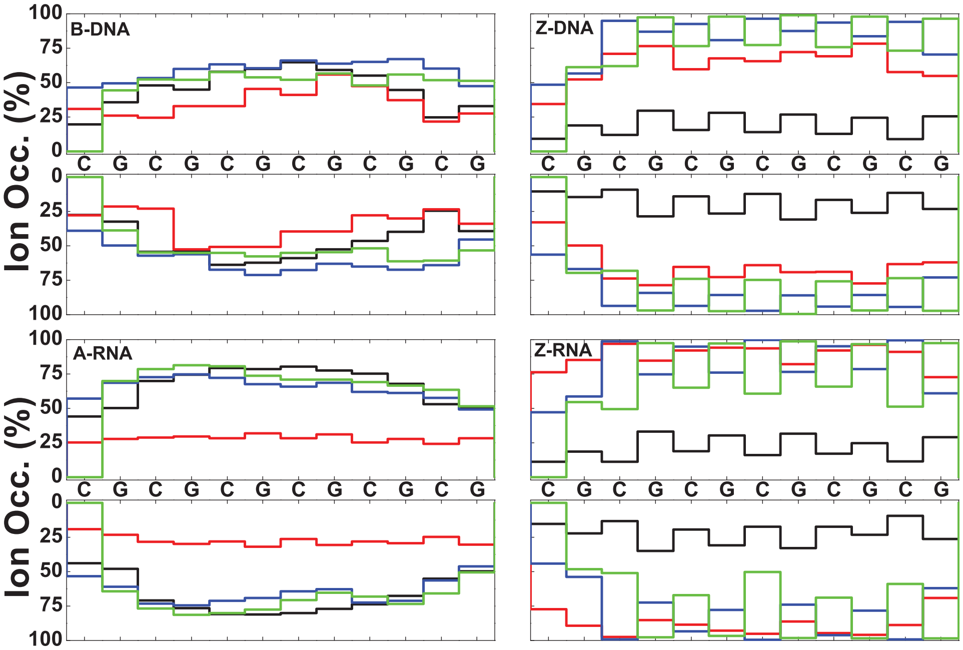


**Supplementary Figure 5. Na$^+$ ion occupancies within 6 Å as function of sequence for 0.4M salt concentration.** Colors represent: major groove (black), minor groove (red), O' oxygen atoms on backbone (blue), and phosphate oxygens (green).

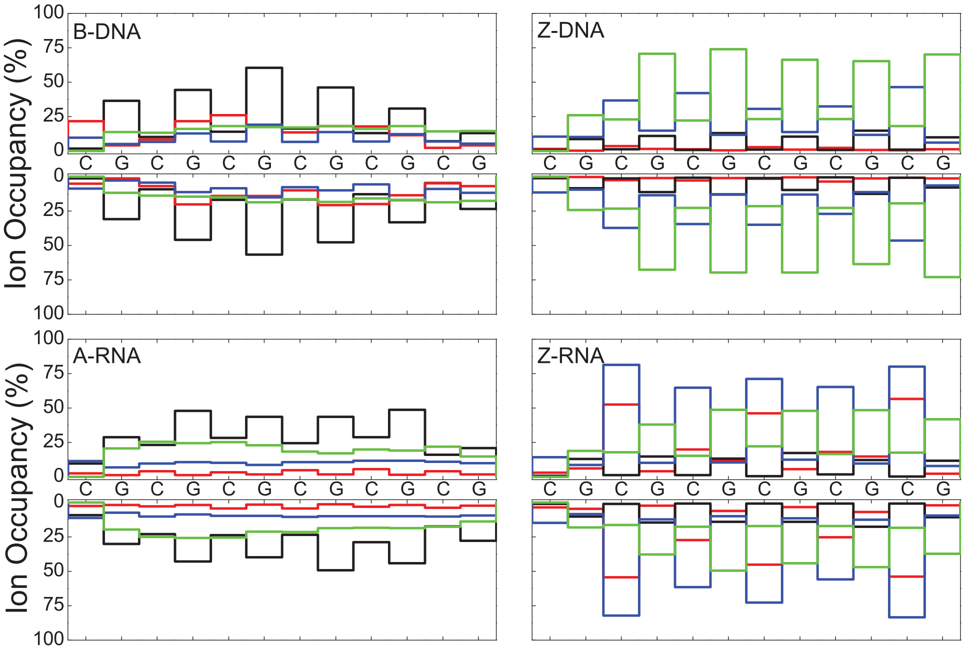


**Supplementary Figure 6. $K^+$ ion occupancies within 3.5 Å as function of sequence for 0.4M salt concentration.** Colors represent: major groove (black), minor groove (red), O' oxygen atoms on backbone (blue), and phosphate oxygens (green).

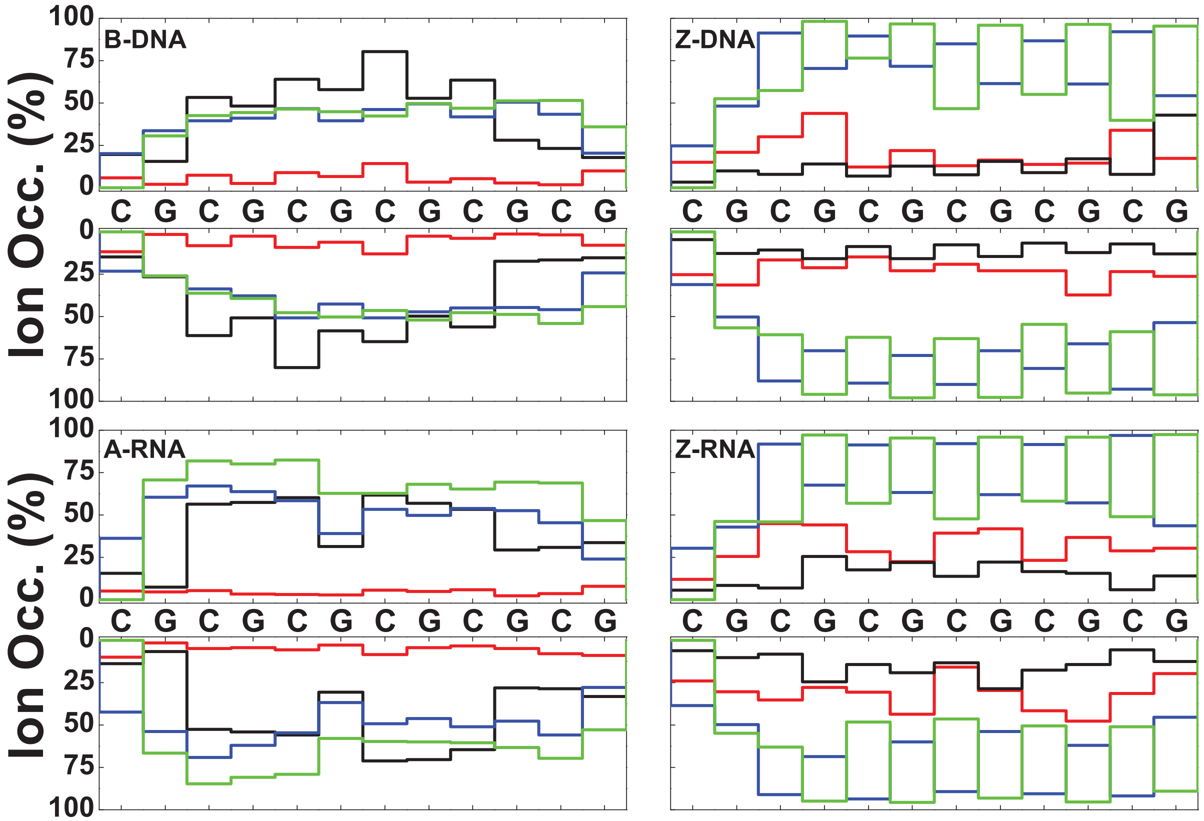


**Supplementary Figure 7. $Mg^{2}+$ ion occupancies within 6 Å as function of sequence for 0.2 M $MgCl_2$ salt concentration.** Colors represent: major groove (black), minor groove (red), O' oxygen atoms on backbone (blue), and phosphate oxygens (green).

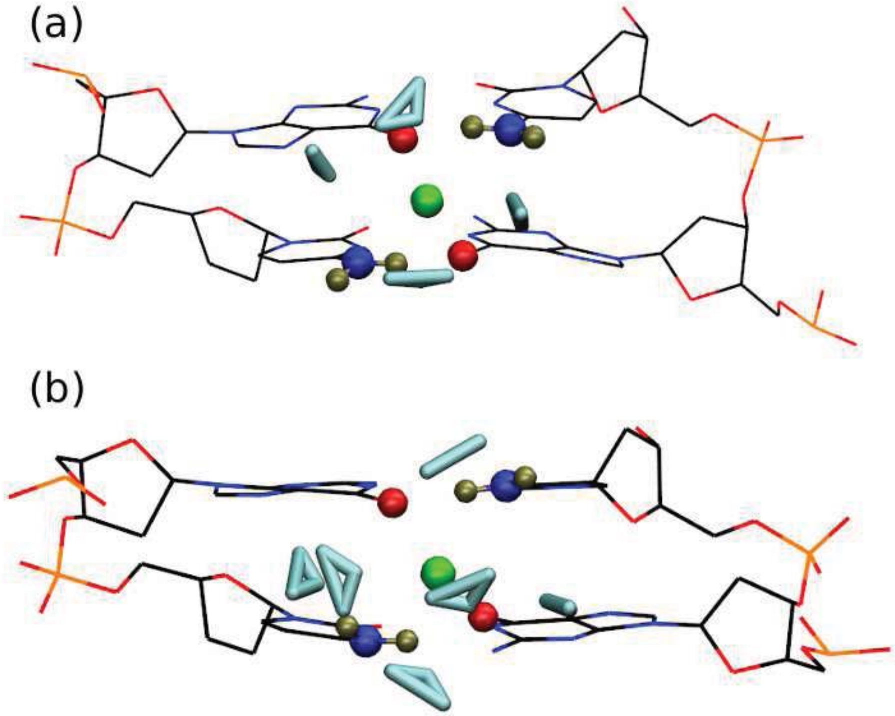


**Supplementary Figure 8. Atomic details of direct binding of monoatomic ions in a "G-O6 – C-N4" configuration.** These snapshots correspond to configurations similar to those in the main text, Fig. 14 a,b. Color representations: O6 (red), N4 (blue), N4-H (tan), water molecules (cyan) and ion (green). (a) $Na^+$ and (b) $K^+$, both in B-DNA at 0.4 M salt concentration. The nearby water molecules are within 3.5Å for $Na^+$ and 4.0Å for $K^+$.

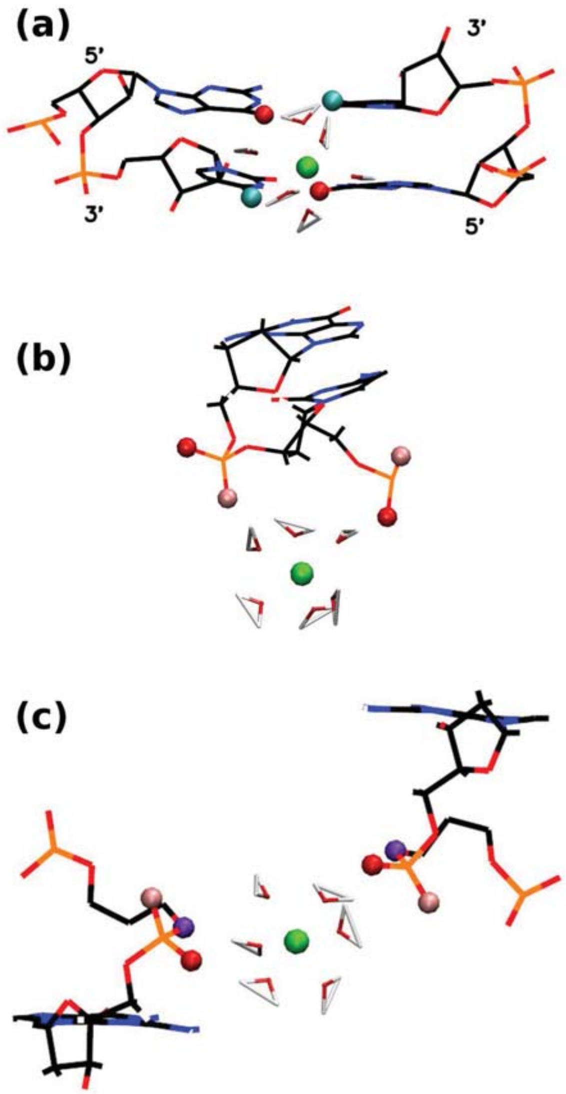


**Supplementary Figure 9. Atomistic details of binding sites of hexahydrated $Mg^{2+}$ (green) for different nucleic acid structures.** (a) Binding to G-O6 (red) in A-RNA (C-N4 atoms are in cyan); (b) binding to phosphate oxygens OP1 (red) and OP2 (pink) in Z-DNA; (c) binding to G-OP1 (red), G-OP2 (pink) and C-O3'(violet) in a CpG step in Z-DNA. The configurations shown are just a snapshot at a given time obtained from the MD simulations.

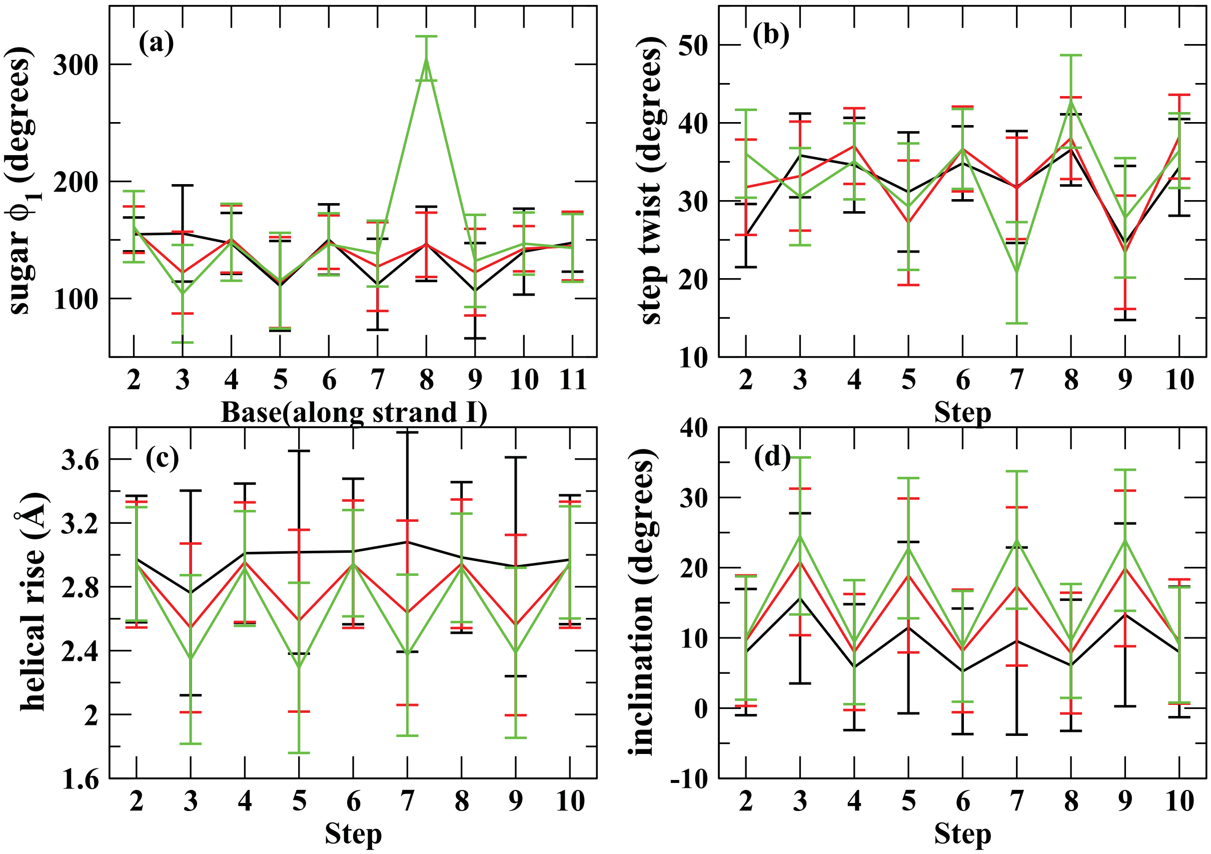


**Supplementary Figure 10. Variation of some nucleic acid parameters for different NaCl excess salt concentrations.** Zero excess salt (black), 0.4 M (red), 4 M (green). The top panels are for B-DNA and the bottom panels for A-RNA. (a) sugar phase angle $\phi_1$ of B-DNA; (b) step twist of B-DNA; (c) helical rise of A-RNA; (d) helical inclination of A-RNA.

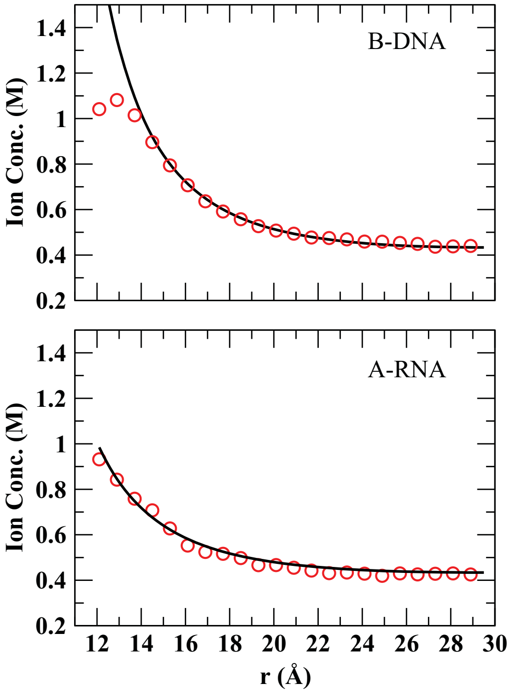


**Supplementary Figure 11. Fitting results of the linear Poisson-Boltzmann theory to the cylindrical ion distribution (CDF) for $Na^+$ at 0.4 M.** The red circles represent the simulation results, while the black curve represents the solution of the continuum theory based on the linear Poisson-Boltzamann model [20]. As may be expected, good fits are obtained away from the central axis.

# References


[1] Case, D. A., Darden, T. A., Cheatham III, T. E., Simmerling, C. L., Wang, J., Duke, R. E., Luo, R., Walker, R. C., Zhang, W., Merz, K. M., Roberts, B. P., Hayik, S., Roitberg, A., Seabra, G., Swails, J., , Kolossvai, A. W. G. I., Wong, K. F., Paesani, F., Vanicek, J., Wolf, R. M., Liu, J., Wu, X., Brozell, S. R., Steinbrecher, T., Gohlke, H., Cai, Q., Ye, X., Wang, J., Hsieh, M. J., Cui, G., Roe, D. R., Mathews, D. H., Seetin, M. G., Salomon-Ferrer, R., Sagui, C., Babin, V., Luchko, T., Gusarov, S., Kovalenko, A., and Kollman, P. A. (2012) "AMBER 12", University of California, San Francisco.

[2] Perez, A., Marchan, I., Svozil, D., Sponer, J., Cheatham, T. E., Laughton, C. A., and Orozco, M. (2007) Refinement of the AMBER Force Field for Nucleic Acids: Improving the Description of $\alpha/\gamma$ Conformers. *Biophys. J.,* **92**, 3817–3829.

[3] Banas, P., Hollas, D., Zgarbva, M., Jurecka, P., Orozco, M., Cheatham, T. E., Sponer, J., and Otyepka, M. (2010) Performance of Molecular Mechanics Force Fields for RNA Simulations: Stability of UUCG and GNRA Hairpins. *J. Chem. Theory Comput.,* **6**, 3836–3849.

[4] Zgarbova, M., Otyepka, M., Sponer, J., Mladek, A., Banas, P., Cheatham, T. E., and Jurecka, P. (2011) Refinement of the Cornell et al. Nucleic Acids Force Field Based on Reference Quantum Chemical Calculations of Glycosidic Torsion Profiles. *J. Chem. Theory Comput.,* **7**, 2886–2902.

[5] Jorgensen, W. L., Chandrasekhar, J., Madura, J., and Klein, M. L. (1983) Comparison of Simple Potential Functions for Simulating Liquid Water. *J. Chem. Phys.,* **79**, 926 – 935.

[6] Joung, I. S. and Cheatham III, T. E. (2008) Determination of Alkali and Halide Monovalent Ion Parameters for use in Explicitly Solvated Biomolecular Simulations. *J. Phys. Chem. B,* **112**, 9020–9041.

[7] Aqvist, J. (1990) Ion-water interaction potentials derived from free energy perturbation simulations. *J. Phys. Chem.,* **94**, 8021 – 8024.

[8] Essmann, U., Perera, L., Berkowitz, M. L., Darden, T., Lee, H., and Pedersen, L. G. (1995) A smooth particle mesh Ewald method. *J. Chem. Phys.,* **103**, 8577 – 8593.

[9] Lu, X. J. and Olson, W. K. (2003) 3DNA: a software package for the analysis, rebuilding and visualization of three-dimensional nucleic acid structures. *Nucleic Acids Res.,* **31**, 5108 – 5121.

[10] Arnott, S. and Hukins, D. W. L. (1972) Optimised parameters for A-DNA and B-DNA. *Biochem. Bioph. Res. Co.,* **47**, 1504–1509.

[11] Arnott, S., Hukins, D., Dover, S., Fuller, W., and Hodgson, A. (1973) Structures of Synthetic Polynucleotides in the A-RNA and A'-RNA Conformations. X-ray Diffraction Analyses of the Molecule Conformations of (Polyadenylic acid) and (Polyi-nosinic acid).(Polycytidylic acid).. *J. Mol. Biol.,* **81**, 109–122.

[12] Moradi, M., Babin, V., Roland, C., and Sagui, C. (2012) Reaction path ensemble of the B-Z-DNA transition: a comprehensive atomistic study. *Nucleic Acids Res.,* **41**, 33–43.

[13] Popenda, M., Milecki, J., and Adamiak, R. W. (2004) High salt solution structure of a left-handed RNA double helix. *Nucleic Acids Res.,* **32**, 4044.

[14] Lu, X. J., Hassan, M. A. E., and Hunter, C. A. (1997) Structure and Conformation of Helical Nucleic Acids: Analysis Program (SCHNAaP). *J. Mol. Biol.,* **273**, 668–680.

[15] Rosenberg, J. M., Seeman, N. C., Day, R. O., and Rich, A. (1976) RNA Double Helices Generated From Crystal-Structures of Double Helical Dinucleoside Phosphates. *Biochem. Bioph. Res. Co.,* **69**, 979–987.

[16] Várnai, P. and Zakrzewska, K. (2004) DNA and its counterions: a molecular dynamics study. *Nucleic Acids Res.,* **320**, 4269–4280.

[17] Atkins, P. W. (1998) Physical Chemistry, Oxford University Press, Oxford, .

[18] Manning, G. S. (1978) Molecular Theory of Polyelectrolyte Solutions With Applications to Electrostatic Properties of Polynucleotides. *Q. Rev. Biophys.,* **11**, 179–246.

[19] Chen, A. A., Marucho, M., Baker, N. A., and Pappu, R. V. (2009) Simulations of RNA Interactions with Monovalent Ions. *Method. Enzymol.,* **469**, 411–432.

[20] Kirmizialtin, S. and Elber, R. (2010) Computational Exploration of Mobile Ion Distributions Around RNA Duplex. *J. Phys. Chem. B,* **114**, 8207–8220.
